# The transcription factor Sox7 modulates endocardiac cushion formation contributed to atrioventricular septal defect through Wnt4/Bmp2 signaling

**DOI:** 10.1038/s41419-021-03658-z

**Published:** 2021-04-12

**Authors:** Nanchao Hong, Erge Zhang, Huilin Xie, Lihui Jin, Qi Zhang, Yanan Lu, Alex F. Chen, Yongguo Yu, Bin Zhou, Sun Chen, Yu Yu, Kun Sun

**Affiliations:** 1 Department of Pediatric Cardiology, Xinhua Hospital, Shanghai Jiao Tong University School of Medicine, 200092 Shanghai, China; 2 Department of Cardiology, Shanghai Institute of Cardiovascular Disease, Zhongshan Hospital, Fudan University, 200032 Shanghai, China; 3 Institute for Developmental and Regenerative Cardiovascular Medicine, Xinhua Hospital, School of Medicine, Shanghai Jiao Tong University, 200092 Shanghai, China; 4 Department of Pediatric Endocrinology and Genetic Metabolism, Shanghai Institute for Pediatric Research, Xinhua Hospital, Shanghai Jiao Tong University School of Medicine, 200092 Shanghai, China; 5 Institute of Biochemistry and Cell Biology, Shanghai Institutes for Biological Sciences, Chinese Academy of Sciences, 200031 Shanghai, China

**Keywords:** Disease model, Epithelial-mesenchymal transition

## Abstract

Cardiac septum malformations account for the largest proportion in congenital heart defects. The transcription factor *Sox7* has critical functions in the vascular development and angiogenesis. It is unclear whether *Sox7* also contributes to cardiac septation development. We identified a de novo 8p23.1 deletion with *Sox7* haploinsufficiency in an atrioventricular septal defect (AVSD) patient using whole exome sequencing in 100 AVSD patients. Then, multiple *Sox7* conditional loss-of-function mice models were generated to explore the role of *Sox7* in atrioventricular cushion development. *Sox7* deficiency mice embryos exhibited partial AVSD and impaired endothelial to mesenchymal transition (EndMT). Transcriptome analysis revealed BMP signaling pathway was significantly downregulated in *Sox7* deficiency atrioventricular cushions. Mechanistically, *Sox7* deficiency reduced the expressions of *Bmp2* in atrioventricular canal myocardium and *Wnt4* in endocardium, and *Sox7* binds to *Wnt4* and *Bmp2* directly. Furthermore, WNT4 or BMP2 protein could partially rescue the impaired EndMT process caused by *Sox7* deficiency, and inhibition of BMP2 by Noggin could attenuate the effect of WNT4 protein. In summary, our findings identify *Sox7* as a novel AVSD pathogenic candidate gene, and it can regulate the EndMT involved in atrioventricular cushion morphogenesis through Wnt4–Bmp2 signaling. This study contributes new strategies to the diagnosis and treatment of congenital heart defects.

## Introduction

Congenital heart defects (CHDs) are the most common form of birth defects, account for ~1% of newborns, and are the leading cause of infant death resulting from birth abnormality, with 22.5% deaths of infants^1^. Cardiac malformations which are attributable to aberrant development of the cardiac septum are the most common CHDs^2,3^. Among them, atrioventricular septal defect (AVSD) covers a spectrum of heart anomalies with a common atrioventricular connection and has an incidence of 4–5.3 per 10,000 live birth^4–6^. A key process in atrioventricular septal development is the endothelial to mesenchymal transition (EndMT), which consists of multiple cellular events including the delamination of endocardial cells from the atrioventricular canal (AVC) endocardium, the acquisition of mesenchymal phenotypes and their invasion into the extracellular matrix (ECM)^7–9^. Further proliferation and remodeling of these mesenchymal cells, as well as the extracellular matrix, result in the formation of membranous portions of the atrial and ventricular septae, and the generation of thin, pliable valves, creating the partitioned four-chamber heart^10^. However, the underlying genetic etiology of AVSD still remains poorly understood.

The SOX family (Sry-related high mobility group box) of transcription factors have pivotal function in multiple developmental processes^11^. *Sox7* is one of the Sox-F gene family (Sox7, Sox17, and Sox18), which plays a critical role in hematopoiesis, angiogenesis, vascular development, and formation^12–17^. In addition, global knockout of *Sox7* is embryonic lethal from E10.5 with developmentally delayed embryos characterized by dilated pericardial sacs and a failure to remodel yolk sac vasculature^18^. However, the role of *Sox7* in mammal heart development still has not been explored.

In this study, we identified an AVSD patient with *Sox7* haploinsufficiency using whole exome sequencing in 100 AVSD patients. Then, multiple *Sox7* conditional loss-of-function mice models were generated to explore the role of *Sox7* in atrioventricular cushion development. *Sox7* endocardial deficiency resulted in impaired atrioventricular cushion EndMT process, which led to improper atrioventricular cushion formation and partial AVSD. We also found that *Sox7* modulates the atrioventricular cushion EndMT process through Wnt4–Bmp2 signaling. This study provides the first evidence that *Sox7* has a critical role in mammal heart development, and contributes new strategies for the diagnosis and treatment of CHDs.

## Results

### Identification of 8p23.1 deletion in an AVSD patient

**Fig. 1:**
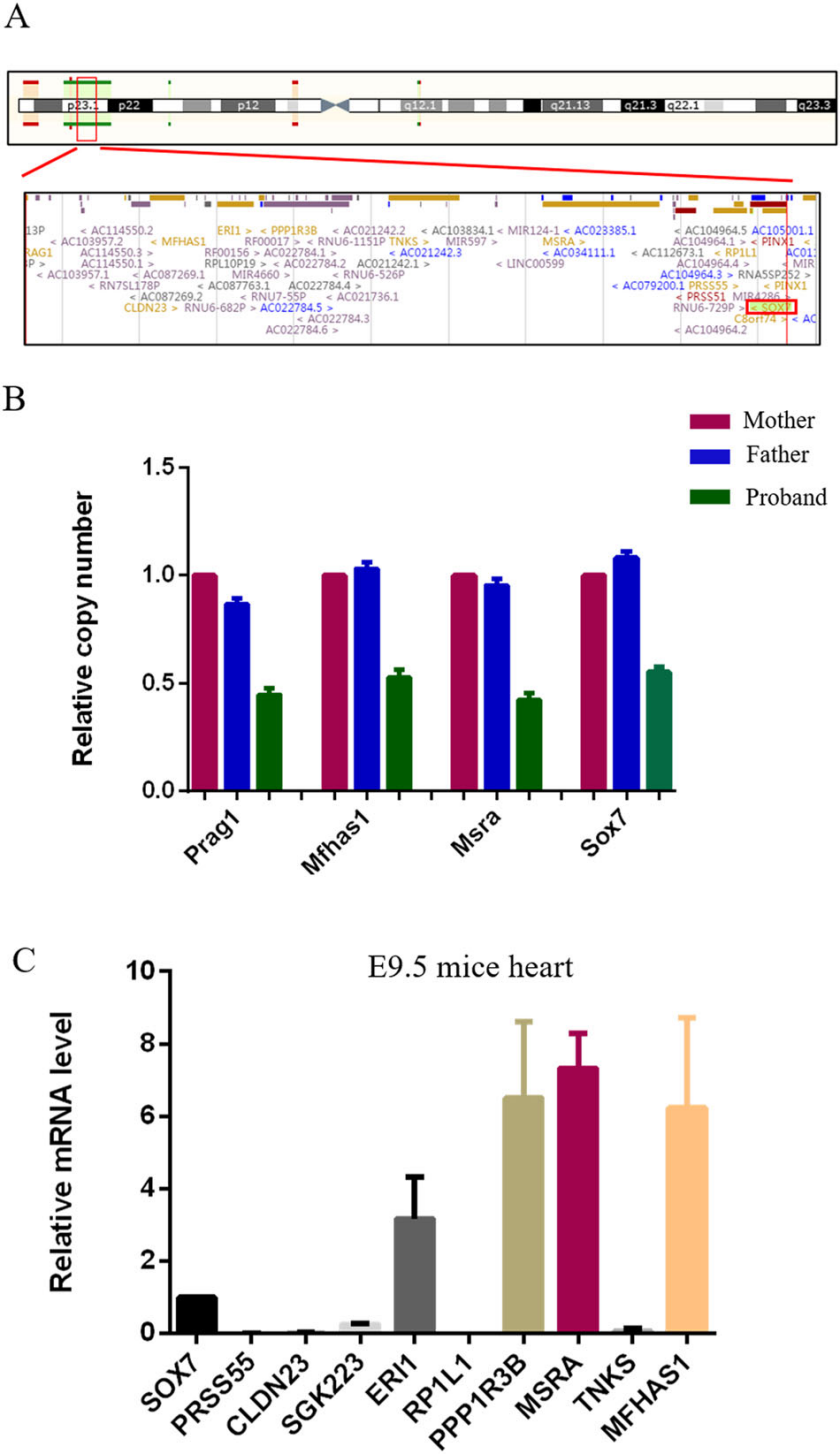
Identification of *Sox7* deficiency in an AVSD patient. **A** The ~2.5 Mb deletion region on chromosome 8p23.1 of the atrioventricular septal defect (AVSD) patient, which contains the *Sox7* gene. **B** qPCR verification showed that the 8p23.1 deletion in the proband was not inherited from parents. **C** the mRNA expression of genes within the 2.5 Mb deletion in mice embryo hearts at E9.5 detected by qPCR (*n* = 3).

We performed whole exome sequencing in 100 Chinese Han AVSD patients and 474 healthy controls. Based on the criteria mentioned in the section “Materials and methods”, we identified 10 rare copy number variants (CNVs) in 13 patients with AVSD finally (Supplementary Table S1). However, among these 10 rare CNVs, those known genes involved in human development of AVSD^19^, including *Gata4*, *Gata5*, *Gata6*, *Nkx2.5*, *Zic3*, *Tbx5*, *Nodal*, *Cfc1*, *Lefty2*, *Acvr2b*, *Bmp4*, *Acvr1*, *Vegf*, *Col6a1-2*, *Fbln2*, *Frzb*, *Dscam*, *Creld1*, *Gja1*, or *Nr2f2* have not been found. Interestingly, we identified a patient with 8p23.1 deletion which was absent from controls, and this 2.5 Mb region did not contain the known pathogenic *Gata4* gene, but contains the *Sox7* gene (Fig. 1A). This patient is a 2-year-old girl, and her clinical diagnosis is partial AVSD, mitral regurgitation (MR) and tricuspid regurgitation (TR), there are no extra cardiac abnormalities. We also collected blood samples from her healthy parents. Verification by qPCR showed that the 8p23.1 deletion of the AVSD patient is de novo (Fig. 1B). Because there were 12 genes in this 2.5 Mb deletion region, we performed qPCR to detect the expression of these genes in mice embryo hearts at E9.5, and found a relatively high expression level of *Sox7*, *Eri1*, *Ppp1r3b*, *Msra*, and *Mfhas1* (Fig. 1C).

### Sox7-deficient mice exhibited abnormal atrioventricular cushions formation

**Fig. 2:**
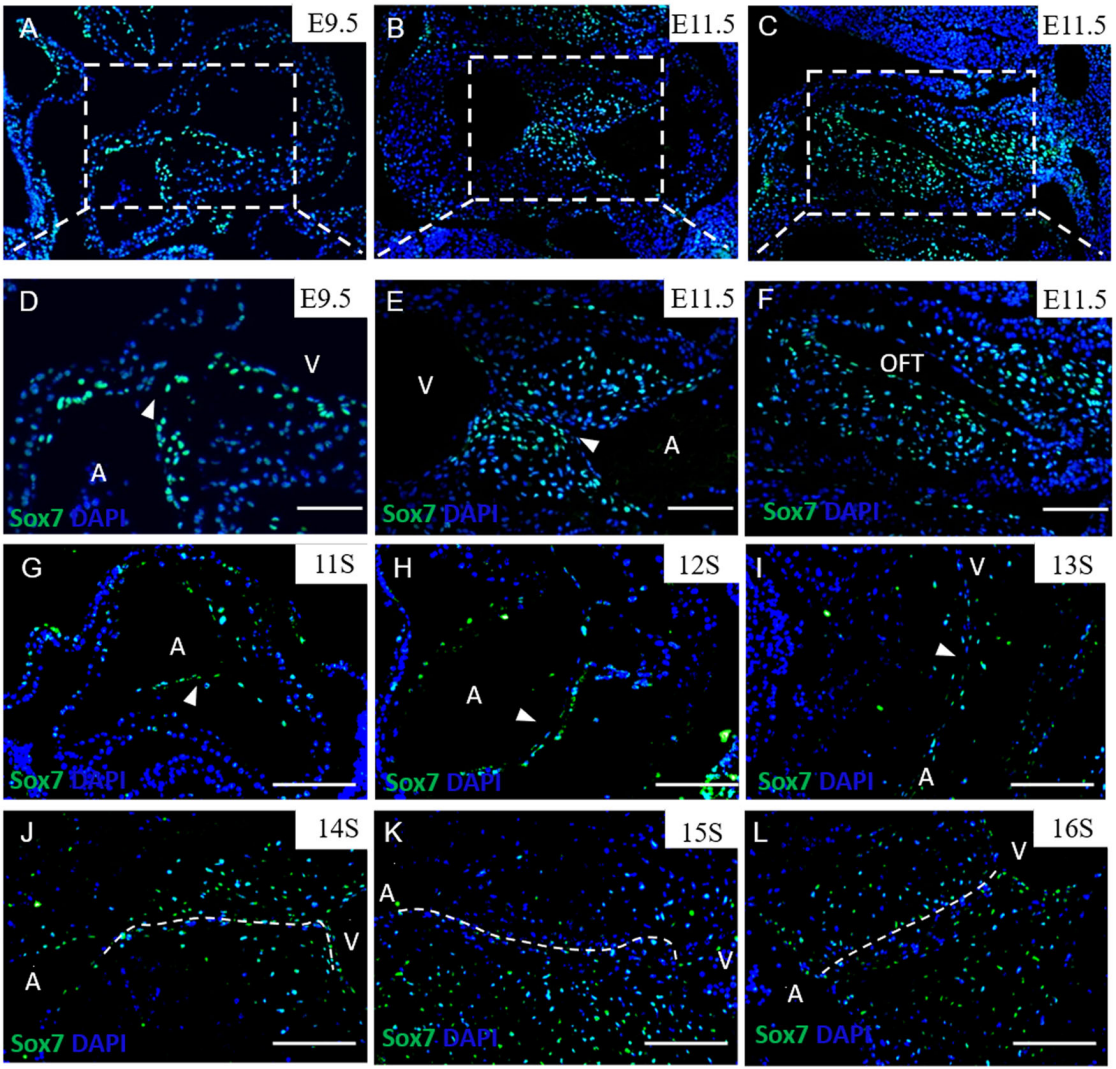
Expression patterns of *Sox7* in mice and human embryos. **A–F** Expression patterns of *Sox7* in mice embryos. *Sox7* was mainly expressed in endocardial cells in the atrioventricular canal (AVC, arrowheads) and the delaminating cells forming the cushion mesenchyme of mice embryos at E9.5 and E11.5, *Sox7* was mainly expressed in both atrioventricular cushion and outflow tract cushion at E11.5 tested by immunofluorescence. **G–L** Expression patterns of *Sox7* in human embryos. SOX7 proteins were strongly expressed in the endocardium overlaying atrioventricular cushion (arrowheads and white dashed lines) and mesenchymal cells derivated from the endocardial cells in the atrioventricular cushion in human embryos at 11S–16S determined by immunofluorescence. A atria, V ventricle, OFT outflow tract. Scale bars: 200 μm.

The heart expression patterns of *Sox7* in mice and human have not been fully explored previously. In mice, we firstly found that *Sox7* was mainly expressed in endocardial cells in the AVC and the delaminating cells forming the cushion mesenchyme at E9.5 (Fig. 2A, D). At E11.5, *Sox7* was mainly expressed in both atrioventricular cushion and outflow tract cushion using immunofluorescence (Fig. 2B, C, E, F). At postnatal stages, *Sox7* was highly expressed in the heart by qPCR (Supplementary Fig. S1A). In human embryos, we found that SOX7 proteins had high abundance in the endocardium overlaying atrioventricular cushion, and mesenchymal cells derivated from the endocardial cells by immunofluorescence (Fig. 2G–L). We also detected *Sox7* mRNA expression in several cell lines by qPCR, including human umbilical vein endothelial cell (HUVEC), human vascular smooth muscle cell (hVSMC), mouse aortic endothelial cell (MAEC), and mouse cardiac muscle cell (HL-1). It demonstrated that *Sox7* was expressed in MAEC and HUVEC, but hardly expressed in hVSMC and HL-1 cell lines (Supplementary Fig. S1B). These data indicated that *Sox7* was strongly expressed in the developing atrioventricular cushion in both mice and human, suggesting the potential role of *Sox7* in cardiac septation.

**Fig. 3:**
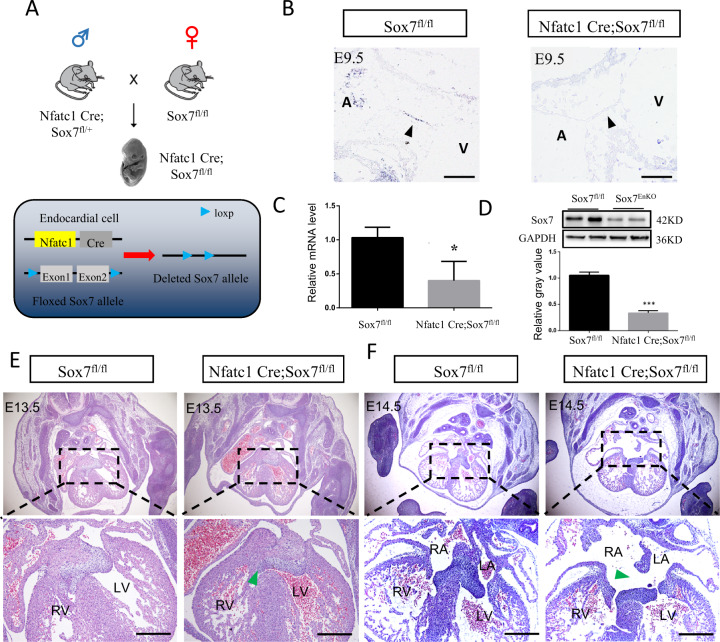
*Sox7*-deficient mice displayed abnormal atrioventricular cushion formation. **A** Schematic diagram showing the generation of *Sox7* deletion in endocardial cells by Nfatc1 Cre. **B** *Sox7* mRNA level was reduced in endocardial cells (arrowheads) in Nfatc1 Cre;Sox7^fl/fl^ embryos at E9.5 detected by in situ hybridization (*n* = 3). **C** *Sox7* mRNA expression of Sox7^fl/fl^ and Nfatc1 Cre;Sox7^fl/fl^ atrioventricular cushions at E9.5 determined by qPCR (*n* = 3). **D** SOX7 protein expression in Sox7^fl/fl^ and Nfatc1 Cre;Sox7^fl/fl^ hearts at E10.5 tested by western blot. The grayscale value of the WB protein bands was calculated using Image J software, *n* = 3. **E** Hematoxylin and eosin (H&E)-stained sectioned hearts illustrated delay in fusion of ventricular septum (VS) with cardiac cushion (CC) in Nfatc1 Cre;Sox7^fl/fl^ embryos at E13.5. The majority of control hearts (Sox7^fl/fl^) exhibited a fully developed VS fused with the CC (left panels); 63.6% (7/11) of Nfatc1 Cre;Sox7^fl/fl^ hearts showed a complete lack of contact between VS and CC (right panels, green arrowhead). **F** H&E-stained sectioned hearts showed defects in closure of the atrial septum (ASD) in Nfatc1 Cre;Sox7^fl/fl^ embryos at E14.5 (*n* = 9). A Atria, V Ventricle, LA left atrium, LV left ventricle, RA right atrium, RV right ventricle. Data are means ± SEM. *P < 0.05, ** *P* < 0.01. Scale bars: 200 μm.

Previous studies have shown that global and endothelial-specific deletion of *Sox7* resulted in embryonic lethality with severely impaired angiogenesis and cardiovascular failure^12,18^. In this study, we firstly inactivated a conditional *Sox7* floxed allele in endocardial lineage cells using Nfatc1-Cre (Fig. 3A). We found Nfatc1 Cre;Sox7^fl/fl^ mice survive after birth. To measure the efficiency of *Sox7* inactivation, we tested its expression in Nfatc1 Cre;Sox7^fl/fl^ and Sox7^fl/fl^ (littermate control) embryos at E9.5 using in situ hybridization, and found that *Sox7* mRNA was reduced in atrioventricular cushions of Nfatc1 Cre;Sox7^fl/fl^ embryos compared with those in controls (Fig. 3B). We also collected atrioventricular cushions and hearts of mice embryos at E9.5 and E10.5, respectively, and detected *Sox7* mRNA and protein by qPCR and western blot. It showed reduced levels of *Sox7* mRNA (by ~50%) and SOX7 protein (by ~70%) in Nfatc1 Cre;Sox7^fl/fl^ samples (Fig. 3C, D).

We then evaluated the phenotypes of Nfatc1 Cre;Sox7^fl/fl^ embryos. At E13.5, 63.6% (7/11) of Nfatc1 Cre;Sox7^fl/fl^ embryos showed incomplete fusion or developmental delay of contact of the developing ventricular septum with the cardiac cushion, compared with 14.3% (1/7) of control embryos (Fig. 3E). In addition, we also found defects in closure of the atrial septum (ASD) at E14.5 in 44.4% (4/9) Nfatc1 Cre;Sox7^fl/fl^ mice (Fig. 3F). These data implicated that *Sox7* deficiency in the endocardium resulted in abnormal cardiac septation.

### Sox7 was required for EndMT process

**Fig. 4:**
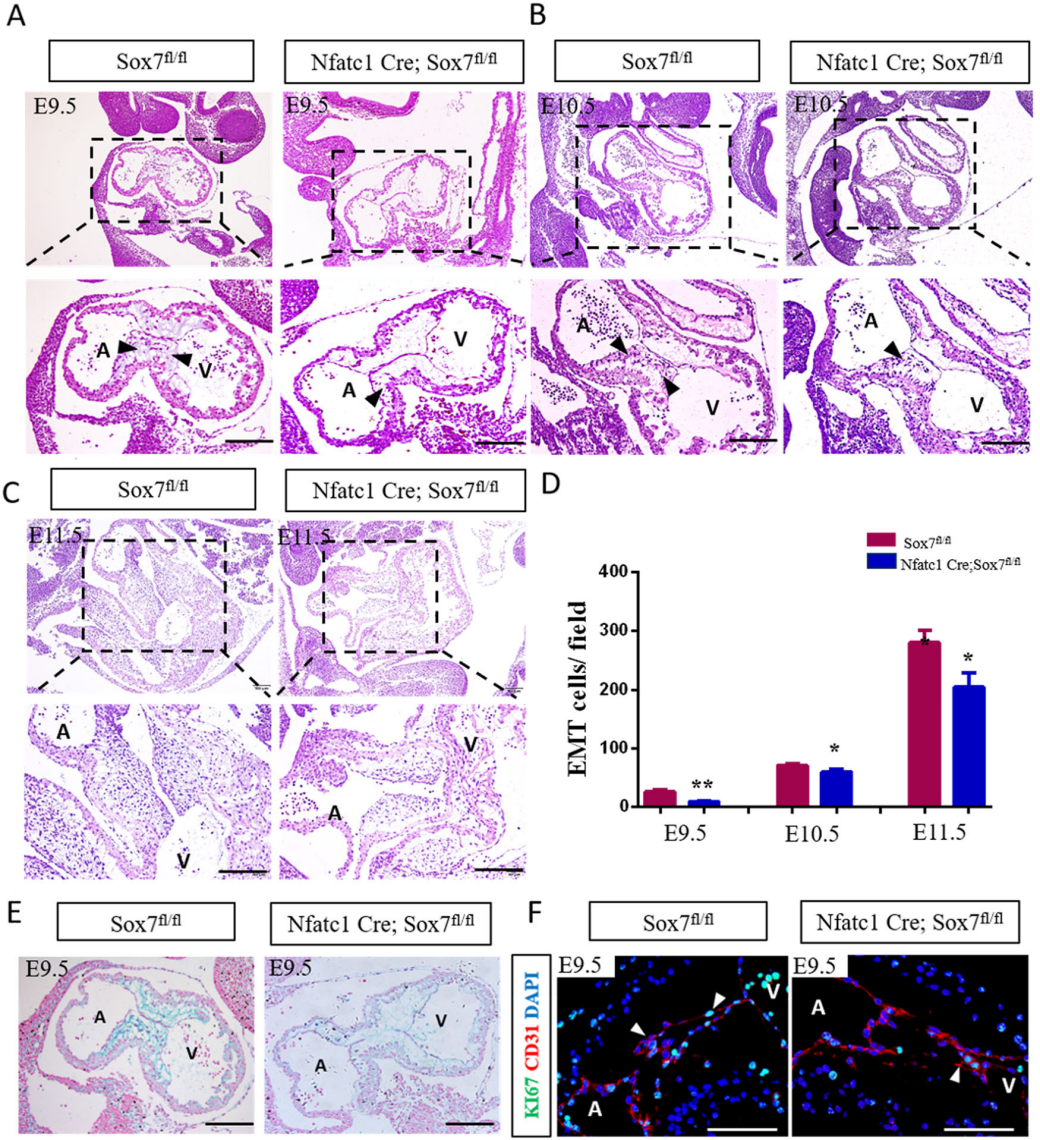
*Sox7* was required for endothelial to mesenchymal transition process in vivo. **A** H&E-stained sections of the E9.5 hearts (*n* = 6). Atrioventricular cushions of control hearts (left panels) were populated by mesenchymal cells formed by endothelial to mesenchymal (EndMT), but there were significantly reduced numbers of mesenchymal cells in atrioventricular cushions of and Nfatc1 Cre;Sox7^fl/fl^ embryos at E9.5. Arrowheads indicated invading mesenchymal cells. **B** H&E-stained sections of the controls (left panels) and Nfatc1 Cre;Sox7^fl/fl^ (right panels) at E10.5 (*n* = 4). Arrowheads indicated invading mesenchymal cells. **C** H&E-stained sections of the controls (left panels) and Nfatc1 Cre;Sox7^fl/fl^ (right panels) at E11.5 (*n* = 4). **D** Quantifications of the number of transforming cells in H&E sections from controls and Nfatc1 Cre;Sox7^fl/fl^ hearts at E9.5, E10.5, and E11.5. **E** Alcian Blue staining showed significantly reduced glycosaminoglycans in atrioventricular cushions of Nfatc1 Cre;Sox7^fl/fl^ embryos at E9.5 (*n* = 4). **F** KI67 staining of E9.5 Nfatc1 Cre;Sox7^fl/fl^ and littermate controls (*n* = 4). Arrowheads indicated KI67 positive endocardial cells. A atrium; V ventricle. Data are means ± SEM. *P < 0.05, **P < 0.01. Scale bars: 200 μm.

A key event in atrioventricular cushion formation is EndMT, which consists of multiple cellular events. To determine the contribution of *Sox7* to EndMT, we bred Sox7^fl/fl^ mice with endocardial lineage-specific Nfatc1-Cre transgenic driver line, endothelium-specific Tie2-Cre transgenic driver line, and cardiac progenitor cell-specific Nkx2.5-Cre transgenic driver line. At E10.5, we found evidence of delayed development and failure of yolk sac vascular remodeling in Tie2 Cre;Sox7^fl/fl^ embryos (Supplementary Fig. S2), confirming previous studies^12^. Furthermore, atrioventricular cushions were populated by mesenchymal cells formed by endocardial EndMT in control hearts, but there were significantly reduced mesenchymal cell numbers in atrioventricular cushions of Nfatc1 Cre;Sox7^fl/fl^ embryos at E9.5, E10.5, and E11.5 (Fig. 4A–D), which pointed out disruption of the EndMT process. We also carefully counted the somite of Nfatc1 Cre; Sox7^fl/fl^ embryos, there was no significant difference between Nfatc1 Cre;Sox7^fl/fl^ embryos and littermate controls at E9.5 (19.80 ± 0.66 vs. 18.75 ± 0.75; *P* = 0.33), therefore, the EndMT defects could be explained by a specific effect of loss of *Sox7* deficiency in the endocardial cells. In addition, we also found significantly reduced mesenchymal cell numbers in atrioventricular cushions Nkx2.5 Cre;Sox7^fl/fl^ embryos at E9.5 (Supplementary Fig. S3A and 3B).

Proper formation of ECM is a crucial step for endocardial EndMT process^20^. Our data showed significantly decreased glycosaminoglycans in atrioventricular cushions of Nfatc1 Cre;Sox7^fl/fl^ embryos at E9.5 using Alcian Blue staining (Fig. 4E), suggesting that *Sox7* deficiency in the endocardium did affect ECM formation. Since proliferation contributes to EndMT and *Sox7* is an important regulator of cell proliferation^21^, we examined the effect of *Sox7* inactivation on proliferation of endocardial cells using immunofluorescence, and found that the number of KI67-positive endocardial cells in Nfatc1 Cre;Sox7^fl/fl^ cushions decreased sharply compared with those in controls (Fig. 4F). Similarly, the number of proliferating cell nuclear antigen (PCNA)-positive endocardial cells in Nfatc1 Cre;Sox7^fl/fl^ cushions was much fewer than those in controls (Supplementary Fig. S3C). However, apoptosis analysis by TUNEL showed that there was no significant difference of endocardial cell apoptosis between Nfatc1 Cre;Sox7^fl/fl^ embryos and controls at E9.5, E11.5 and E14.5 (Supplementary Fig. S3D, E and F). We then studied whether *Sox7* promotes proliferation by regulating cell cycle factors, the mRNA expression levels of cell cycle factors (Cyclin a2, Cyclin d2, Cdkn1a, Cdkn2b, and Cdk4) were detected in hearts of Nfatc1 Cre;Sox7^fl/fl^ embryos and control embryos at E9.5, and we found reduced expression of Cyclin a2 and Cyclin d2 in mutant embryos compared with the control ones (Supplementary Fig. S4A). To explore whether *Sox7* can regulate cell cycle, we overexpressed *Sox7* in mouse embryonic endocardial cell (MEEC) cell lines, and the flow cytometry results suggested the overexpression group had a significant higher proliferation index than that in the control group (Supplementary Fig. S4B and C). These findings indicated that endocardial ablation of Sox7 resulted in inhibited endocardial cells proliferation, which likely contributed to the hypocellular cushion defects in Nfatc1 Cre;Sox7^fl/fl^ embryos.

**Fig. 5:**
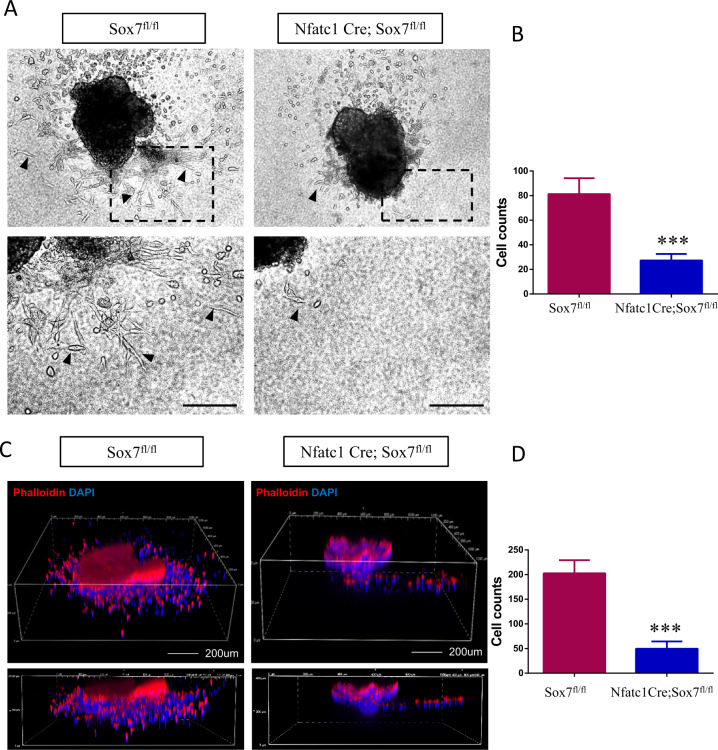
*Sox7* was required for endothelial to mesenchymal transition process in vitro. **A** Representative AVC explants of Nfatc1 Cre;Sox7^fl/fl^ and littermate control at E9.5 after 72 h in culture. Arrowheads indicated migrating cells and spindle-shaped cells. **B** Quantification of the numbers of cells that migration over the top of matrix from control (*n* = 5) and Nfatc1 Cre;Sox7^fl/fl^ AVC explants (*n* = 4). **C** Three-dimensional reconstruction of mutant explant and control one. **D** Quantification of the numbers of cells that migrated and invaded into the matrix from control (*n* = 4) and Nfatc1 Cre;Sox7^fl/fl^ AVC explants (*n* = 4). Data are means ± SEM. ***P < 0.001. Scale bars: 200 μm.

To further identify whether *Sox7* was required for endocardial EndMT, we also performed in vitro collagen gel assays and calculated the numbers of mesenchymal cells that have migrated from the explant into the matrix after 72 h. We found significantly reduced migration over the top of matrix in Nfatc1 Cre;Sox7^fl/fl^ AVC explants compared with those in littermate control (Sox7^fl/fl^) AVC explants (27.25 ± 2.66 vs. 81.25 ± 6.46, *P* < 0.001) (Fig. 5A, B). In particular, we performed 3D reconstruction using NIS-Elements software (Nikon) to show cells that migrated away from explants and invaded the gel visually, and we found significantly reduced migration and invasion in Nfatc1 Cre;Sox7^fl/fl^ AVC explants compared with those in littermate control (Sox7^fl/fl^) AVC explants (49.75 ± 7.32 vs. 202.5 ± 13.42, *P* < 0.001) (Fig. 5C, D). These data indicated that *Sox7* was required for normal endocardial EndMT process. Impaired EndMT process might elucidate abnormal atrioventricular cushion formation in *Sox7*-deficient mice.

### **Decreased BMP signaling in AVC of*Sox7***-deficient embry**os**

**Fig. 6:**
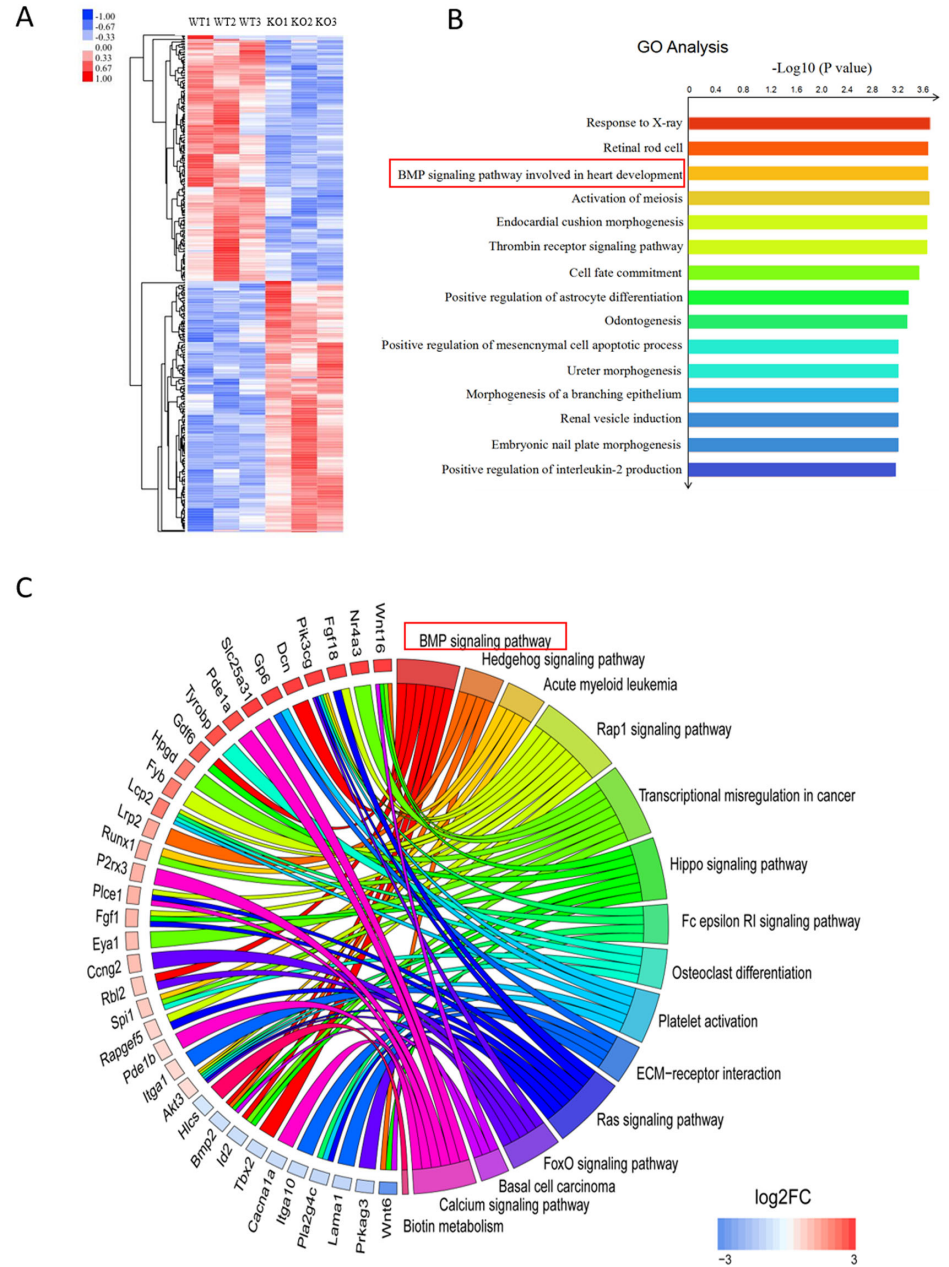
Transcriptome analysis identified the downstream target genes of *Sox7* in the atrioventricular cushion formation. **A** Heatmap of the differentially expressed genes (DEGs) identified by comparing the transcriptomes of AVCs from control (Sox7^fl/fl^) and Nfatc1 Cre;Sox7^fl/fl^ (KO) mouse embryonic hearts at E9.5 (three biological replicates were analyzed). DEGs were genes whose expression were significantly changed (≥1.5-fold) between control and Sox7-deficient samples (*P* < 0.05). **B** GO-enrichment analysis for biological processes for the 152 downregulated and 218 upregulated genes. **C** Circular plot representing 36 differentially expressed genes from 15 pathways identified by Ingenuity pathway analysis. The color code for DEG levels (bottom right) was represented as a logarithmic of fold change (logFC): red indicated upregulated, blue was downregulated, and gray delegated unchanged. Decreased BMP Signaling pathway in AVCs of Nfatc1 Cre;Sox7^fl/fl^ embryos were highlighted by red boxes.

Then, we screened altered signaling pathways underlying the hypocellular atrioventricular cushion phenotype of Nfatc1 Cre;Sox7^fl/fl^ embryos using an RNA-sequencing (RNA-seq) detection in microdissected AVC tissues of E9.5 Nfatc1 Cre;Sox7^fl/fl^ embryos and controls (Sox7^fl/fl^). The transcriptome analysis yielded 370 differentially expressed genes of which 218 were upregulated and 152 downregulated (Fig. 6A; Supplementary Fig. S5A). Functional analysis of the differentially expressed genes identified several Gene Ontology (GO) categories, and the most highly represented GO term was Response to X-ray, followed by Retinal rod cell, BMP signaling pathway involved in heart development, activation of meiosis, and endocardial cushion morphogenesis (Fig. 6B; Supplementary Fig. S5B). Ingenuity pathway analysis figured out 15 altered pathways (Fig. 6C; Supplementary Fig. S5C).

**Fig. 7:**
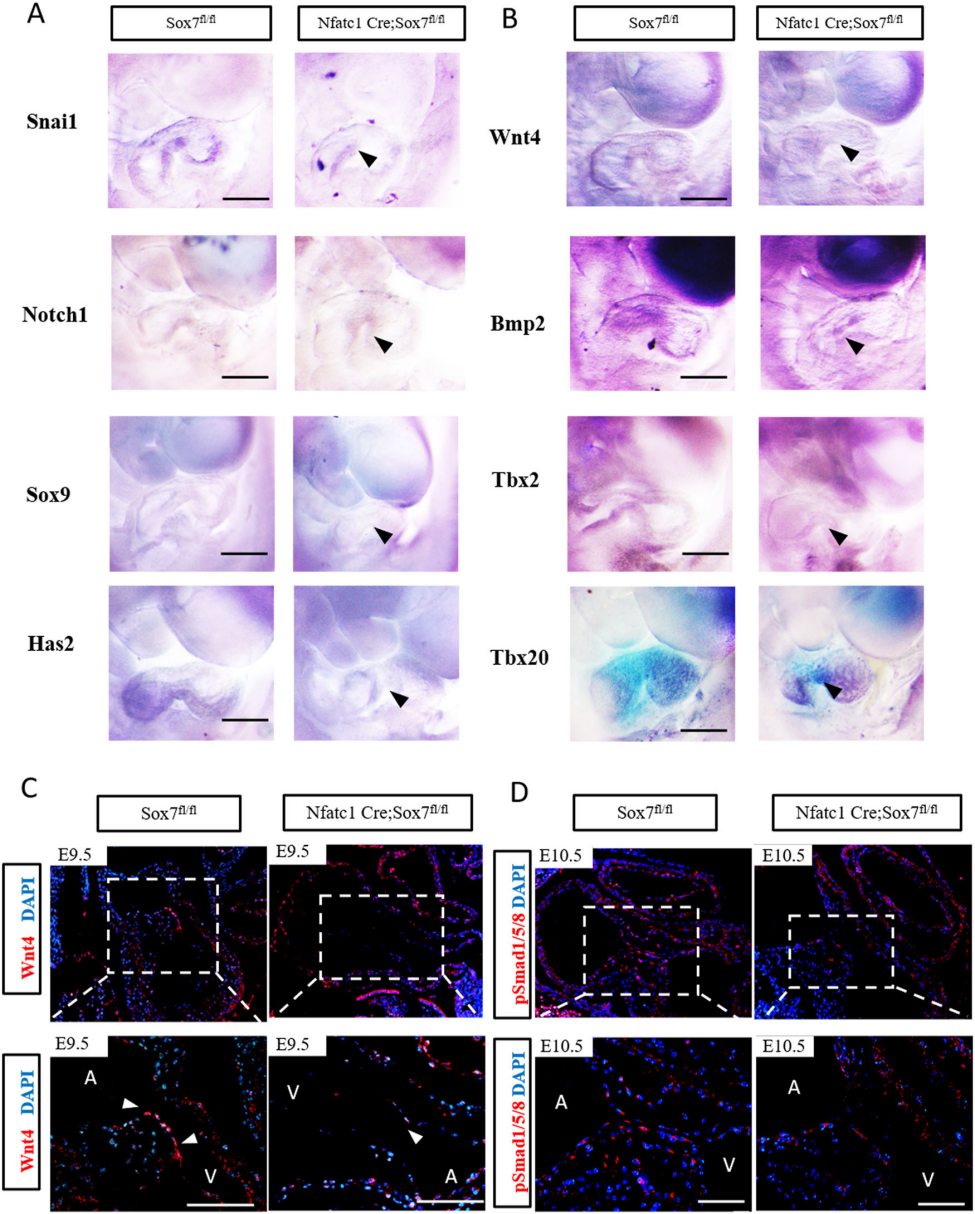
Spatial expression changes in some of the differentially expressed *Sox7* downstream genes verified by whole-mount in situ hybridization and immunofluorescence. **A** Atrioventricular cushion EndMT markers *Snai1*, *Sox9*, and *Has2* were reduced in Nfatc1 Cre;Sox7^fl/fl^ AVCs compared with those in littermate controls (Sox7^fl/fl^) at E9.5, whereas *Notch1* expression remained unchanged, which was in agreement with the results of RNA-seq analysis. Arrowheads indicated Nfatc1 Cre;Sox7^fl/fl^ AVCs (*n* = 4). **B** *Wnt4* expression in endocardium, *Bmp2* and its target *Tbx2* expression in AVC myocardium were reduced in Nfatc1 Cre;Sox7^fl/fl^ AVCs compared with those in littermate controls (Sox7^fl/fl^) at E9.5, whereas *Tbx20* expression remained unchanged. Arrowheads indicated Nfatc1 Cre;Sox7^fl/fl^ AVCs (*n* = 4). **C** Immunofluorescence confirmed the reduced expression of WNT4 in the AVC endocardium of Nfatc1 Cre;Sox7^fl/fl^ at E9.5. Arrowheads indicated *Wnt4* positive endocardial cells (*n* = 4). **D** Immunofluorescence revealed significantly decreased levels of phosphorylated-Smad1/5/8 (pSmad1/5/8) in atrioventricular cushions of E10.5 Nfatc1 Cre;Sox7^fl/fl^ embryos compared with those in controls (*n* = 4). A atrium, V ventricle. Scale bars: 200 μm.

Next, we utilized whole-mount in situ hybridization (WISH) and immunofluorescence to further test the expression alterations of those markers which related to atrioventricular cushion development selected from the RNA-seq results. We found that the expression of *Snai1* which is a key regulator of EndMT was downregulated in Nfatc1 Cre;Sox7^fl/fl^ AVCs (Fig. 7A), and we also found increased expression of VE-cadherin which was directly repressed by *Snai1* in EndMT in AVCs of Nfatc1 Cre;Sox7^fl/fl^ embryos compared with that in controls at E9.5 (Supplementary Fig. S6A). However, another EndMT regulator, *Notch1*, was not changed in Nfatc1 Cre;Sox7^fl/fl^ AVCs compared with that in controls (Fig. 7A). We also examined the transcription factors *Sox9* and *Msx1*, which are expressed in the atrioventricular cushion mesenchyme and crucial for EndMT process^22,23^. It showed that both of them were reduced in Nfatc1 Cre;Sox7^fl/fl^ AVCs compared with those in controls (Fig. 7A; Supplementary Fig. S6B). Moreover, in agreement with the Alcian Blue staining data (Fig. 4E), the expression of *Has2* which is responsible for deposition of the major glycosaminoglycan of the atrioventricular cushion, was also decreased in atrioventricular cushions of Nfatc1 Cre;Sox7^fl/fl^ embryos compared with that in controls (Fig. 7A).

Pathway analysis of the RNA-seq data identified significant reduction of the BMP/TGF-beta signaling pathway in Nfatc1 Cre;Sox7^fl/fl^ AVCs (Fig. 6C; Supplementary Fig. S5C). We further revealed that the expression of *Bmp2* in atrioventricular cushion myocardium was downregulated in Nfatc1 Cre;Sox7^fl/fl^ AVCs by WISH (Fig. 7B), but another factor *Tgfβ-2* which is also expressed in atrioventricular cushion myocardium was grossly normal in Nfatc1 Cre;Sox7^fl/fl^ AVCs compared with controls (Supplementary Fig. S6B). In particular, *Tbx2*, a direct target of Bmp2 signaling pathway during atrioventricular cushion development^24^, was also reduced in Nfatc1 Cre;Sox7^fl/fl^ AVCs (Fig. 7B). As well known, the Bmp2 signaling pathway works through phosphorylation and nuclear localization of the Smad1/5/8 complex. Interestingly, we also found that phosphorylated-Smad1/5/8 (pSmad1/5/8) levels in atrioventricular cushions of E10.5 Nfatc1 Cre;Sox7^fl/fl^ embryos were decreased compared with those in controls using immunofluorescence (Fig. 7D). However, *Bmp2* is mainly expressed in AVC myocardium, we then pondered over how endocardial *Sox7* regulated the AVC myocardium *Bmp2* expression. Wnt and Bmp, two critical signaling pathways that have already been shown to regulate EndMT in AVC endocardium and myocardium, respectively^25^. Therefore, we overexpressed *Sox7* in the MEEC line to explore several Wnt ligands expressions, and it showed that only *Wnt4* was significantly changed (Supplementary Fig. S7A and B). Moreover, *Wnt4*, which is expressed in endocardium, can regulate *Bmp2* expression in AVC myocardium during atrioventricular cushion development^26^. So, we measured the *Wnt4* expression in Nfatc1 Cre;Sox7^fl/fl^ embryos, it showed that *Sox7* deficiency reduced *Wnt4* expression in endocardium, as measured by both WISH and immunofluorescence (Fig. 7B, C). In addition, *Tbx20*, an upstream transcription factor of *Bmp2* remained unchanged in Nfatc1 Cre;Sox7^fl/fl^ AVCs (Fig. 7B), indicating that *Sox7* regulated Bmp2 signaling independent of *Tbx20*. WISH analysis of another transcription factor *Pitx2*, whose transcript levels were significantly altered in Nfatc1 Cre;Sox7^fl/fl^ AVCs by RNA-seq, showed no changes between Nfatc1 Cre;Sox7^fl/fl^ embryos and controls (Supplementary Fig. S6B).

All these findings indicated that *Sox7* affected endocardial EndMT process and atrioventricular cushion development via Wnt4 and Bmp2 signaling.

### ***Sox7*transcriptionally regulates Wnt4–Bmp2 signaling**

**Fig. 8:**
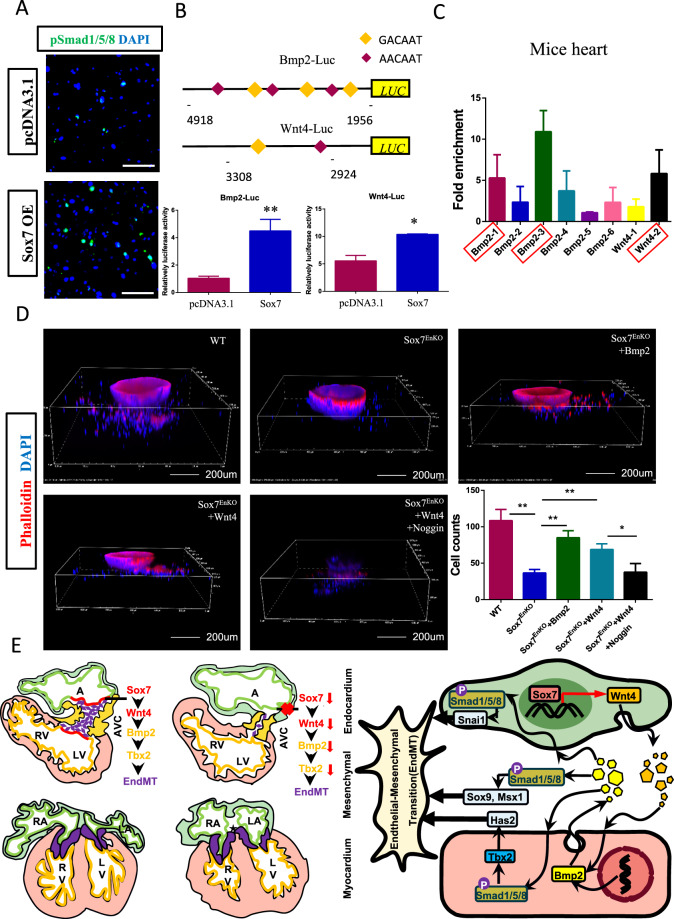
*Sox7* transcriptionally regulated Wnt4–Bmp2 signaling. **A** Immunofluorescence of pSmad1/5/8 in *Sox7*-overexpression and wild type MEEC. **B** The schematic diagram of Sox7-binding sites in the promoter regions of *Bmp2* and *Wnt4*, and the effect of *Sox7* overexpression on the luciferase activity of *Bmp2* and *Wnt4* promoter in MEEC (*n* = 3). **C** Chromatin immunoprecipitation (ChIP) assay of adult mice hearts by ChIP-qPCR which showed nucleic acid electrophoresis of genomic DNA fragments after ChIP using anti-Sox7 antibody compared to control isotype for *Bmp2* and *Wnt4* (*n* = 4), the binding sites were highlighted by red boxes. **D** In vitro 3D collagen gel EndMT assays showed that WNT4 or BMP2 recombinant protein additions was able to rescue EndMT defect induced by *Sox7* deficiency, and blocking BMP2 activities with Noggin attenuated above rescue caused by WNT4 protein (*n* = 3). Quantitative analysis of the number of transformed mesenchymal cells under each treatment. Data are means ± SEM. *P < 0.05, **P < 0.01. Scale bars: 200 μm. **E** Schematic illustration of how *Sox7* regulates atrioventricular cushion development through Wnt4–Bmp2 signaling. *Sox7* expression in AVC endocardium drives EndMT via *Wnt4* and *Bmp2* activation in AVC endocardium (red) and myocardium (yellow), respectively, in E9.5 WT heart, and a four-chamber heart is formed eventually at E14.5. *Sox7* deficiency downregulates *Wnt4* and *Bmp2* expression, which disrupts normal EndMT process, leading to abnormal formation of atrioventricular cushion at E14.5. During early atrioventricular cushion development, *Sox7* is mainly expressed in the AVC endocardium, it can activate endocardial *Wnt4* expression directly, which then subsequently acts as a paracrine factor to upregulate *Bmp2* expression in the adjacent AVC myocardium to trigger EndMT via *Tbx2* and *Snail*, *Bmp2* also regulates EndMT through promoting *Sox9*, *Msx1*, and *Twist1* expression in the atrioventricular cushion mesenchyme.

In an attempt to explore the specific modulation between *Sox7* and *Bmp2* signaling, we overexpressed *Sox7* in MEEC, then the expressions of *Wnt4*, *Bmp2* and its target gene *Tbx2* were examined. The mRNA levels of *Wnt4*, *Bmp2*, and *Tbx2* were significantly increased in MEEC (Supplementary Fig. S7A). To determine whether Smad nuclear localization was altered by *Sox7* overexpression, we therefore performed pSmad1/5/8 immunostaining assay in MEEC, and found that *Sox7* overexpression significantly increased pSmad1/5/8 expression and nuclear localization (Fig. 8A). Interestingly, nuclear colocalization of SOX7 and pSmad1/5/8 was also found in the endocardium overlaying atrioventricular cushions of human 13S embryos (Supplementary Fig. S7C). These data suggested that *Sox7* regulated pSmad1/5/8 accumulation in the nucleus. Sequence analysis showed that there were several *Sox7*-binding sites in the promoter regions of *Wnt4* and *Bmp2* (Fig. 8B). Luciferase assays revealed that *Sox7* could activate the promoter of both *Wnt4* and *Bmp2* in MEEC (Fig. 8B). We then performed ChIP-qPCR, and found that *Sox7* bound to the promoter regions of *Wnt4* and *Bmp2* in both adult mice hearts and MEEC cell lines (Fig. 8C and Supplementary Fig. S7D). Specifically, *Sox7* bounds to the first and third *Sox7*-binding site in the promoter of *Bmp2* and the second *Sox7*-binding site in the promoter of *Wnt4* in adult mice hearts, and *Sox7* bounds to the first, third, and sixth *Sox7*-binding site in the promoter of *Bmp2* and the first *Sox7*-binding site in the promoter of *Wnt4* in MEEC cell lines (Fig. 8C and Supplementary Fig. S7E). These data indicated that *Wnt4* and *Bmp2* might be *Sox7* direct downstream target genes. *Bmp2* is an important regulator of mesenchymal cell proliferation during endocardial cushion formation^27,28^, so we examined the mesenchymal cell proliferation by KI67 staining, and found that mesenchymal cell proliferation was depressed in Nfatc1 Cre;Sox7^fl/fl^ AVCs compared with that in controls at E10.5 (Supplementary Fig. S7F).

To determine whether Wnt4–Bmp2 signals were required for the regulation of *Sox7* on EndMT process, we performed rescue experiments using the collagen gel EndMT assay. We cultured AVC explants isolated from E9.5 Nfatc1 Cre;Sox7^fl/fl^ embryos and controls, and found that the migration and invasion by transformed mesenchymal cells in Nfatc1 Cre;Sox7^fl/fl^ AVC explants were inhibited compared with those in controls (Fig. 8D). Nevertheless, the addition of recombinant WNT4 or BMP2 protein was able to partially rescue the EndMT defect caused by *Sox7* deficiency, and blocking BMP2 activities with Noggin attenuated rescue of impaired EndMT by WNT4 protein (Fig. 8D). Taken together, these data implicated that *Sox7* regulated EndMT process through Wnt4–Bmp2 signaling axis (Fig. 8E).

## Discussion

AVSD is a genetically heterogeneous defect. Several syndromes are associated with AVSD, including Down syndrome, Noonan syndrome, Holt–Oram syndrome, and 8p23.1 deletion syndrome^6,29–31^. We used whole exome sequencing to screen out possible pathogenic CNVs in 100 AVSD patients and 474 healthy controls. We identified an AVSD patient harbored a de novo 8p23.1 deletion, it did not contain the *GATA4* gene which is considered as a likely candidate gene for the heart defects of 8p23.1 deletion patient but contained the *Sox7* gene. Similarly, Wat et al. ^32^ indicated that the haploinsufficiency of *Sox7* may increase severity of the cardiac phenotype in 8p23.1 deletion syndrome patient with *GATA4* deletion. We also performed qPCR to detect the expression of genes within the 2.5 Mb deletion in mice embryo hearts at E9.5, and found a relatively high expression level of *Sox7*, *Eri1*, *Ppp1r3b*, *Msra*, and *Mfhas1*. However, there was no report on the relationship between *Eri1* or *Mfhas1* and cardiovascular disease/development. Wei-J et al. reported that *Ppp1r3b* polymorphisms were associated with serum LDL-C levels, the risk of coronary artery disease and ischemic stroke in the Southern Chinese Han population^33^. The lack of *Msra* exacerbates cardiovascular disease phenotypes driven by increased oxidative stress^34^. Nevertheless, only *Sox7* is related to cardiovascular development^12,16,17^. Therefore, we considered *Sox7* as the candidate gene for the AVSD patient in our study.

The role of *Sox7* in mammal heart development has not been explored. In humans, *Sox7* was expressed in the heart, lung, brain, tongue, vertebrae, and liver of the 8-week human embryo^35,36^. We showed here for the first time that SOX7 proteins were strongly expressed in the endocardium overlaying atrioventricular cushion in early human embryos (4–6 weeks). A previous study by deleting *Sox7* in pan-endothelium with Tie2-Cre indicated an essential role of *Sox7* in angiogenesis^12^. However, these *Sox7* mutant embryos died before E10.5. In our study, we deleted *Sox7* specifically in endocardium and its mesenchymal progeny using the Nfatc1 cre driver line, which developed normal yolk sac vasculature and survived till to postnatal stage, allowing us to intensively study the role of *Sox7* in atrioventricular cushion morphogenesis. Histological analysis revealed that the EndMT process of Nfatc1 Cre;Sox7^fl/fl^ and Nkx2.5 Cre;Sox7^fl/fl^ mice embryos were disrupted. Nfatc1 was expressed in the developing heart of mice embryos at E7.75–E9.0^37^, occurred before our proliferation and apoptosis analysis, which allowed us to explore the role of *Sox7* in endocardial cells proliferation and apoptosis at E9.5. We found *Sox7* may promote cell proliferation by upregulating cell cycle factors Cyclin a2 and Cyclin d2, and the reduced cell proliferation is at least one of the reasons that resulted in formation of hypocellular endocardial cushions in Nfatc1 Cre;Sox7^fl/fl^ embryos. In addition, we found incomplete fusion or developmental delay of contact of the developing ventricular septum with the cardiac cushion in Nfatc1 Cre;Sox7^fl/fl^ embryos at E13.5, which was similar to a previous study of *Nipbl* haploinsufficiency mouse model^38^, and we also found defects in closure of the ASD at E14.5 in Nfatc1 Cre;Sox7^fl/fl^ mice. Taken together, all these data pointed out the abnormal formation of cardiac septation in *Sox7*-deficient mice.

The regulation of atrioventricular cushion formation consists of a series of complex and molecular factors. The BMP signaling pathway, particularly *Bmp2*, is a well-known paracrine signal secreted from myocardium for EndMT and post-EndMT mesenchymal cell proliferation during endocardial cushion formation^26,39^. Signaling crosstalk between endocardium and myocardium has been showed essential for atrioventricular cushion EndMT process. It has been reported that Jagged1-Notch1 signaling in endocardial cells can induce the expression of endocardial *Wnt4*, which subsequently acts as a paracrine factor to upregulate *Bmp2* expression in the adjacent AVC myocardium to trigger EndMT^26^. *Wnt4* is a member of the WNT family of secreted molecules that function in a paracrine manner to affect several developmental changes, and *Bmp2* is also the downstream target gene of *Wnt4* during ovary development^40^. In this study, we found *Bmp2* signaling was downregulated in Nfatc1 Cre;Sox7^fl/fl^ AVCs at E9.5 by both RNA-seq and WISH. Furthermore, we firstly showed that *Sox7* deficiency reduced endocardial *Wnt4* expression, luciferase and ChIP-qPCR assays identified *Wnt4* as a direct downstream target of *Sox7*. In addition, WNT4 or BMP2 treatment was able to partially rescue EndMT defect caused by *Sox7* deficiency, and blocking BMP2 activities with Noggin attenuated rescue of impaired EndMT by WNT4. It will be interesting to investigate how endocardial *Wnt4* regulates AVC myocardial *Bmp2* expression in the future.

## Materials and methods

### Mouse models

The conditional Sox7 loss-of-function mice (Sox7^flox/flox^, Sox7^fl/fl^) were obtained from The Jackson Laboratory (USA, Stock No. 027711). We generated Sox7 endocardial lineage-specific conditional knockout by crossing Sox7^fl/fl^ with Nfatc1-Cre mice, Nfatc1-Cre mice have been described elsewhere^41^. Nkx2.5-Cre and Tek-Cre (also known as Tie2-Cre) mice were also provided by The Jackson Laboratory (USA, Stock Nos. 027711 and 008863, respectively).

### Human subjects

Our study recruited 100 AVSD patients in Xinhua Hospital Affiliated to Shanghai Jiao Tong University School of Medicine and Shanghai Children’s Medical Center whose diagnoses were confirmed by echocardiography, cardiac catheterization examinations, computed tomography, and other operation recordings. 474 healthy controls were also enrolled.

### Whole exome sequencing analysis and quantitative real-time polymerase chain reaction validation

We performed whole-exome sequencing in 100 AVSD patients and 474 healthy controls. The DNA was sequenced using the Illumina HiSeq 2500 platform at a commercial provider (Shanghai Biotechnology Co, Ltd., Shanghai, China). The data of CNVs were filtered. We distinguished common CNVs from rare CNVs by comparing the results with the known CNVs in the Database of Genomic Variants (DGV, http://dgv.tcag.ca/) and Online Mendelian Inheritance in Man (OMIM, http://omim.org). Rare CNV segments were identified based on the following criteria: (1) CNV > 500 kb, but <5 Mb in size; (2) average depth of sequencing >54; (3) *Z*-score of average depth ≥2.0; (4) present at <0.1% frequency or not found in the DGV (http://dgv.tcag.ca/). Finally, we identified a patient with 8p23.1 deletion which was absent from controls, and we also collected blood samples from her healthy parents, they were verified by quantitative real-time polymerase chain reaction (qPCR) at a commercial provider (Genesky Biotechnologies Inc., Shanghai). Moreover, we performed qPCR to detect the expression of genes within the 8p23.1 deletion in mice embryo hearts at E9.5. Primers used to amplify genes within the 8p23.1 deletion region are shown in Supplementary Table S2.

### Histology detection

Mouse tissues were fixed with 4% paraformaldehyde in PBS, paraffin embedded, sectioned at 4-μm intervals, and processed. Hematoxylin and eosin (H&E) and Alcian Blue staining were performed using standard procedures. Five embryos were analyzed for H&E and Alcian Blue staining each.

### AVC explant cultures

A 1.5 mg/ml solution of rat-tail collagen type I (BD Biosciences) was dispensed into four-well microculture dishes and allowed to solidify inside a 37 °C, 5% CO_2_ incubator. Collagen gels were washed several times with Opti-MEM. Subsequently, the wells were filled with Opti-MEM containing 1% fetal calf serum, 1% insulin-transferrinseleniun (ITS, Invitrogen), and antibiotics, and incubated overnight. AVC explants from E9.5 embryonic hearts were carefully dissected in ice cold PBS, cut open and placed with the endocardium facing down onto the collagen matrixes after removing the excess medium. Following placement, the explants were left to attach for 24 h at 37 °C in 5% CO_2_. Medium and specific treatments were added to the dishes, and explants were cultured for 3 days, then all cells that migrated away from explants and invaded the gel were counted. For 3D reconstruction of the E9.5 explants, images were taken of several optical sections per explant and reconstructions were performed using NIS-Elements software (Nikon). For rescue experiments, Bmp2 (200 ng/ml) (R&D systems), Wnt4 (250 ng/ml) (Proteintech), and Noggin (200 ng/ml) (Novoprotein) were added to the explant culture. After fixation in 4% paraformaldehyde (30 min room temperature), antigens were detected using Phalloidin-TRITC (1:100, Yeasen Biotech) and nuclei counterstained with DAPI and analyzed using a Leica SP5 confocal microscope. 4–6 explants were analyzed for each group.

### Cell culture and transfection

HUVEC, hVSMC, MAEC, HL-1, and MEEC cell lines were obtained from the Type Culture Collection of the Chinese Academy of Sciences (Shanghai, China) and cultured in the DMEM medium (Invitrogen) supplemented with 10% fetal calf serum (Invitrogen), penicillin (100 units/ml), and streptomycin (100 μg/ml). Cells were incubated at 37 °C in a humid atmosphere with 5% CO_2_. The transient transfection was performed with Fugene HD transfection reagent (Promega) according to the manufacturer’s protocol for adherent cells.

### Flow cytometry cell cycle analysis

MEEC cells were seeded into six-well plates and transfected with control or wild-type Sox7 plasmids. Cells were trypsinized and collected after 48 h. Cells were washed with PBS, fixed in ice-cold 70% ethanol and kept at 4 °C overnight. The following day, cells were resuspended in PBS, centrifuged, and stained with propidium iodide. Flow cytometry analysis was performed using CellQuest software (BD Biosciences, USA). The proliferation index = (G2/M + S)/(G2/M + S + G1/G0).

### Transcriptome analysis

AVCs dissected from Nfatc1 Cre;Sox7^fl/fl^ and control (Sox7^fl/fl^) embryos at E9.5 were flash frozen in RNAlater^TM^ solution (Invitrogene), 10 AVCs were pooled per replicate. The RNA quality was checked by Bioanalyzer 2200 (Aligent) and the RIN > 6.0 is suitable for RNA sequencing. The cDNA libraries were constructed for each pooled RNA sample using the NEBNext^®^ Ultra™ Directional RNA Library Prep Kit for Illumina according to the manufacturer’s instructions.

### In situ hybridization

Whole mount and section in situ hybridization were conducted as described previously^42,43^. Details of probes are shown in Table S3.

### Quantitative real-time polymerase chain reaction

Total RNA from cardiac tissues and cell lines was isolated using TRIzol reagent (Life Technologies), and converts to cDNA using the PrimeScript™ RT Master Mix (Takara). For qPCR, SYBR Green qPCR master mix (Takara) was used following the protocol supplied by the manufacturer. Primers for qPCR are shown in Table S4.

### Luciferase assay

Transfections and dual luciferase reporter assays were carried out as described previously^44^. The luciferase reporters of Bmp2-luc and Wnt4-luc were constructed by inserting the conserved Sox7-binding sites^45^ in the promoter regions of the *Bmp2* and *Wnt4* into the pGL3-promoter plasmid (Promega).

### ChIP-qPCR assays

ChIP-qPCR assays of MEEC line and mice cardiac tissues were performed using the chromatin immunoprecipitation assay kit (Millipore) and EpiQuik Tissue Chromatin Immunoprecipitation Kit (Epigentek) following the protocol supplied by the manufacturer. Primers used for ChIP-qPCR are listed in Supplementary Table S5.

### Immunofluorescence

Immunofluorescence was carried out as described previously^2^. Antibodies used were listed: Sox7 (R&D system, AF2766), pSmad1/5/8 (CST, 13820), Smad1/5/9 (Abcam, ab80255), Wnt4 (Proteintech, 14371-1-AP), CD31 (Wuhan Servicebio Technology, GB11063-2), VE-cadherin (Abcam, ab205336), KI67 (Wuhan Servicebio Technology, GB111141), and PCNA (Wuhan Servicebio Technology, GB11010). For TUNEL staining, the In Situ Cell Death Detection Kit (Roche) was used according to the manufacturer’s instruction. 4–6 embryos were analyzed for each experiment.

### Western blot

Proteins from mice embryos were separated by 10% SDS–PAGE and transferred electrophoretically onto Immobilon-P polyvinylidene difluoride (PVDF) membranes (Millipore). The membranes were probed with Sox7 polyclonal antibody (Proteintech, 23925-1-AP) and GAPDH antibody (Wuhan Servicebio Technology, GB11002) followed by either anti-rabbit (Jackson) or anti-mouse (Jackson) IgG secondary antibodies conjugated to horseradish peroxidase, then detected with a chemiluminescence system (BioRad).

### Statistics

All data are represented as mean (SD) of at least three independent experiments unless otherwise stated in the figure legend, and statistical analysis was performed using the Student’s *t* test or one-way or two-way ANOVA followed by the Bonferroni’s multiple comparison test with GraphPad Prism version 5.0 software. *P* < 0.05 was considered statistically significant.

## Supplementary information

Supplementary Figure legends

Supplementary figure 1

Supplementary figure 2

Supplementary figure 3

Supplementary figure 4

Supplementary figure 5

Supplementary figure 6

Supplementary figure 7

Supplementary Tables
