# Supplementary material for: The transcription factor Sox7 modulates endocardiac cushion formation contributed to atrioventricular septal defect through Wnt4/Bmp2 signaling
Source: Cell Death Dis. 2021 Apr 12;12(4):393. doi: 10.1038/s41419-021-03658-z (PMC8041771; doi:10.1038/s41419-021-03658-z)
Supplement: Supplementary file 1 — Supplementary Figure legends [file 41419_2021_3658_MOESM1_ESM.docx]

**FIGURE LEGENDS**

**Figure S1. Expression patterns of *Sox7* in cardiovascular tissues and cell lines. A,** Relative mRNA expression of *Sox7* in mice heart and aorta detected by qPCR (n=3). **B,** Relative mRNA expression of *Sox7* in HUVEC, hVSMC, MAEC and HL-1 determined by qPCR (n=3).

**Figure S2. Gross morphology of different transgenic mice embryos.** Tie2 Cre;Sox7^fl/fl^ embryos showed evidence of delayed development and failure of yolk sac remodeling at E10.5, while Nfatc1 Cre;Sox7^fl/fl^ and Nkx2.5 Cre;Sox7^fl/fl^ embryos were grossly normal compared with those in controls at E10.5 (n=4).

**Figure S3. Atrioventricular cushion agenesis in *Sox7* deficient mouse embryos. A,** H&E stained sections of E9.5 hearts in Nkx2.5 Cre;Sox7^fl/fl^ embryos (n=4). Atrioventricular cushions of control hearts (left panels) were populated by mesenchymal cells derived from EndMT, but there were significantly reduced mesenchymal cells in atrioventricular cushions of Nkx2.5 Cre;Sox7^fl/fl^ (right panels) embryos. Arrowheads indicated invading mesenchymal cells. **B,** Quantification of the number of transforming cells in H&E sections from control and Nkx2.5 Cre;Sox7^fl/fl^ hearts at E9.5. **C,** PCNA staining of E9.5 Nfatc1 Cre;Sox7^fl/fl^ and littermate controls (n=4). Arrowheads represented PCNA positive endocardial cells. **D, E and F,** Apoptosis of cushion endocardial cells in Nfatc1 Cre;Sox7^fl/fl^ and control hearts at E9.5, E11.5 and E14.5 detected by TUNEL assays. A: atrium; V: ventricle; LA: Left atrium; RA: Right atrium. Data are means±SD. **P*<0.05. Scale Bars: 200μm.

**Figure S4.** Regulation of cell cycle by *Sox7*. A, RT-qPCR was performed to determine the mRNA expression levels of cell cycle factors (Cyclin a2, Cyclin d2, Cdkn1a, Cdkn2b and Cdk4) in hearts of Nfatc1 Cre;Sox7^fl/fl^ embryos and control embryos at E9.5, n=4; B and C, Flow cytometry analysis was performed in MEEC cells transfected with control or wildtype *Sox7* plasmids. Proliferation index = (G2/M+S) /(G2/M+S+G1/G0), n = 3. * *P*<0.05, ** *P*<0.01.

**Figure S5. Transcriptome analysis of AVCs in Nfatc1-Cre;Sox7^fl/fl^ and control groups. A,** Volcano map of the differentially expressed genes (DEGs) identified by comparing the transcriptomes of wild-type (Sox7^fl/fl^) and Nfatc1-Cre;Sox7^fl/fl^ (KO) AVCs from mouse embryonic hearts at E9.5 (three biological replicates were analyzed). DEGs were genes whose expression were significantly changed (≥1.5-fold) between wild-type and Sox7 deficient samples (*P* < 0.05). **B,** Top 20 of GO enrichment analysis for biological processes for the 152 downregulated and 218 upregulated genes. **C,** Top 20 of pathway enrichment analysis for biological processes for the 152 downregulated and 218 upregulated genes.

**Figure S6. Spatial expression changes in some of the differentially expressed Sox7 downstream genes verified by whole-mount in situ hybridization. A,** Immunofluorescence of VE-cadherin in mutant embryos and controls at E9.5. B, The mRNA expression of EndMT marker *Msx1* was reduced in Nfatc1-Cre;Sox7^fl/fl^ AVCs compared with that in littermate controls (Sox7^fl/fl^) at E9.5 (n=3), which was in agreement with the results of RNA-seq analysis. *Tgfβ-2* which is expressed in the atrioventricular cushion myocardium was grossly normal in Nfatc1-Cre;Sox7^fl/fl^ AVCs at E9.5 (n=3). The mRNA expression of *Pitx2* showed no changes between Nfatc1-Cre;Sox7^fl/fl^ AVCs and controls (n=3). Arrowheads indicated Nfatc1-Cre;Sox7^fl/fl^ AVCs. A: atrium; V: ventricle. Scale Bars: 200μm.

**Figure S7. *Sox7* transcriptionally regulated Wnt4-Bmp2 signaling. A,** *Sox7* elevated the mRNA expressions of *Wnt4*, *Bmp2* and *Tbx2* in MEEC by qRT-PCR. **B,** *Sox7* overexpression had no effects on the mRNA expressions of *Wnt2*, *Wnt5a* and *Wnt9a* in MEEC. **C,** Nuclear colocalization of SOX7 and pSmad1/5/8 was found in the endocardium overlaying atrioventricular cushions of human 13S embryos. Arrowhead indicated SOX7 and pSmad1/5/8 positive endocardial cells. **D and E,** Chromatin immunoprecipitation (ChIP) assay of MEEC overexpressing *Sox7*. ChIP-qPCR (right) and in agarose gel (left) (n=4), the effective binding sites were highlighted by red boxes. **F,** Reduced mesenchymal cell proliferation detected by KI67 staining was found in Nfatc1-Cre;Sox7^fl/fl^ atrioventricular cushions compared with that in controls at E10.5 (n=4). Arrowheads indicated KI67 positive cells. Data are means±SD. **P*＜0.05. Scale Bars: 200μm.
