# Supplementary figures and images for: The transcription factor Sox7 modulates endocardiac cushion formation contributed to atrioventricular septal defect through Wnt4/Bmp2 signaling
Source: Cell Death Dis. 2021 Apr 12;12(4):393. doi: 10.1038/s41419-021-03658-z (PMC8041771; doi:10.1038/s41419-021-03658-z)

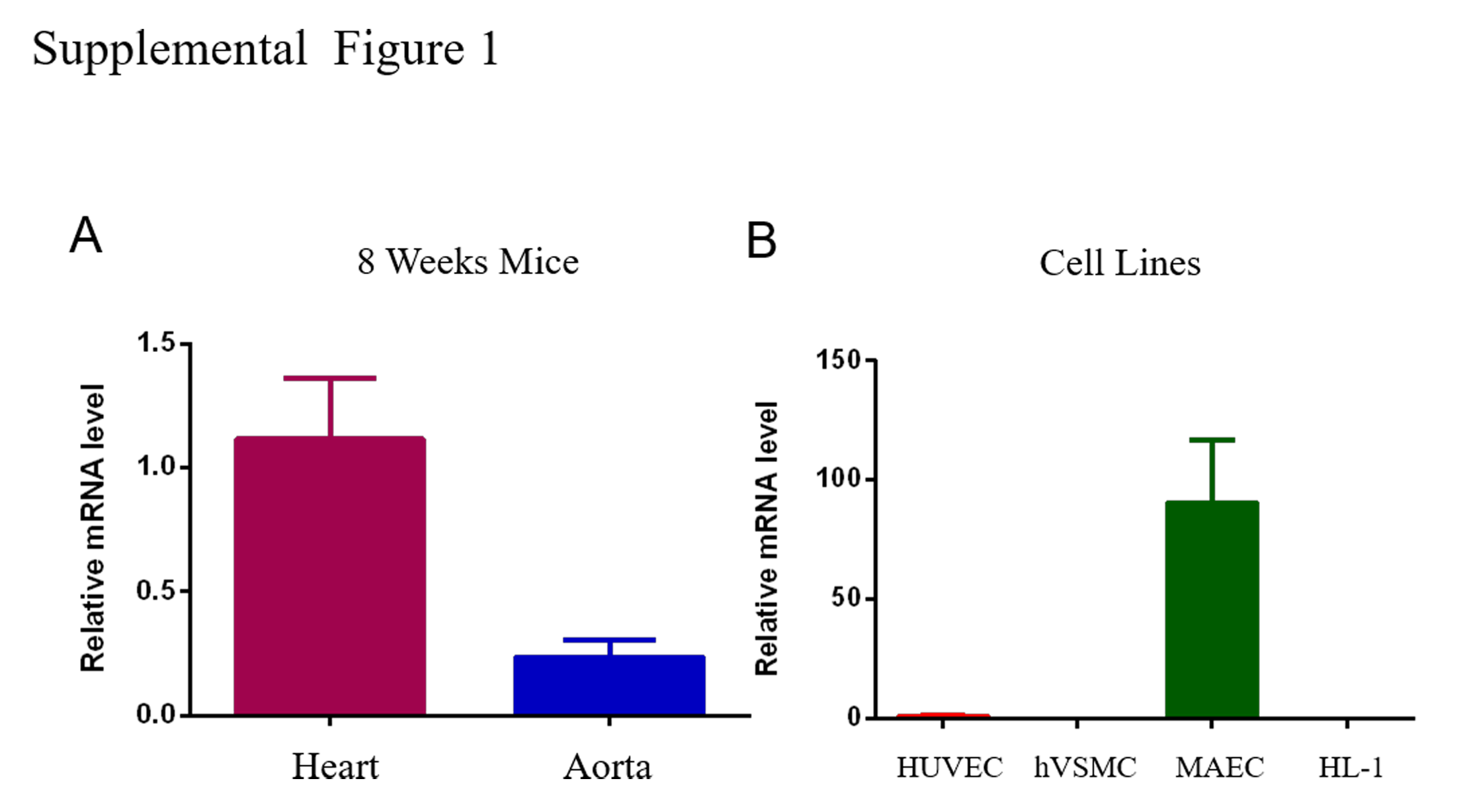

Supplement: Supplementary file 2 — Supplementary figure 1 [file 41419_2021_3658_MOESM2_ESM.tif]

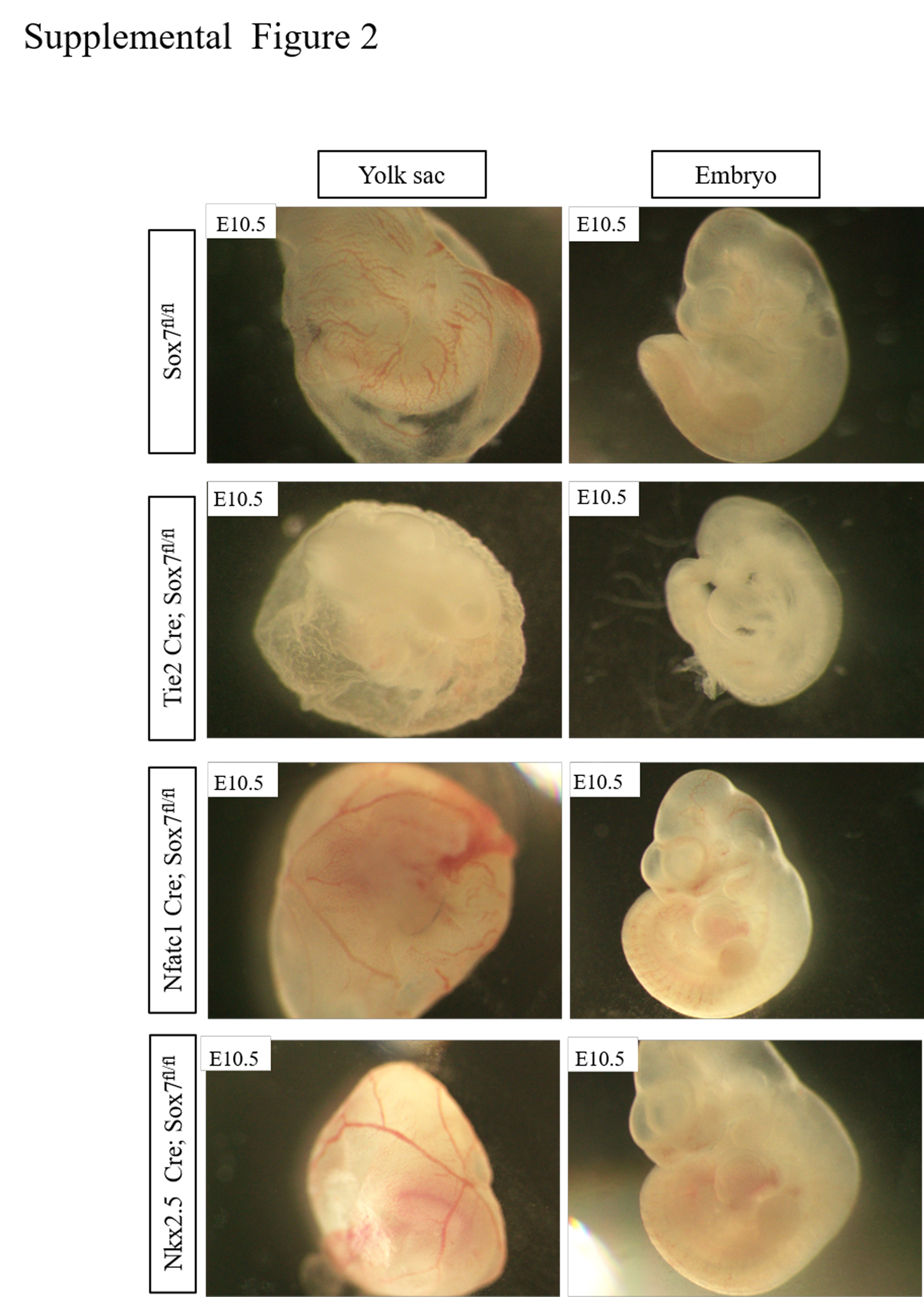

Supplement: Supplementary file 3 — Supplementary figure 2 [file 41419_2021_3658_MOESM3_ESM.tif]

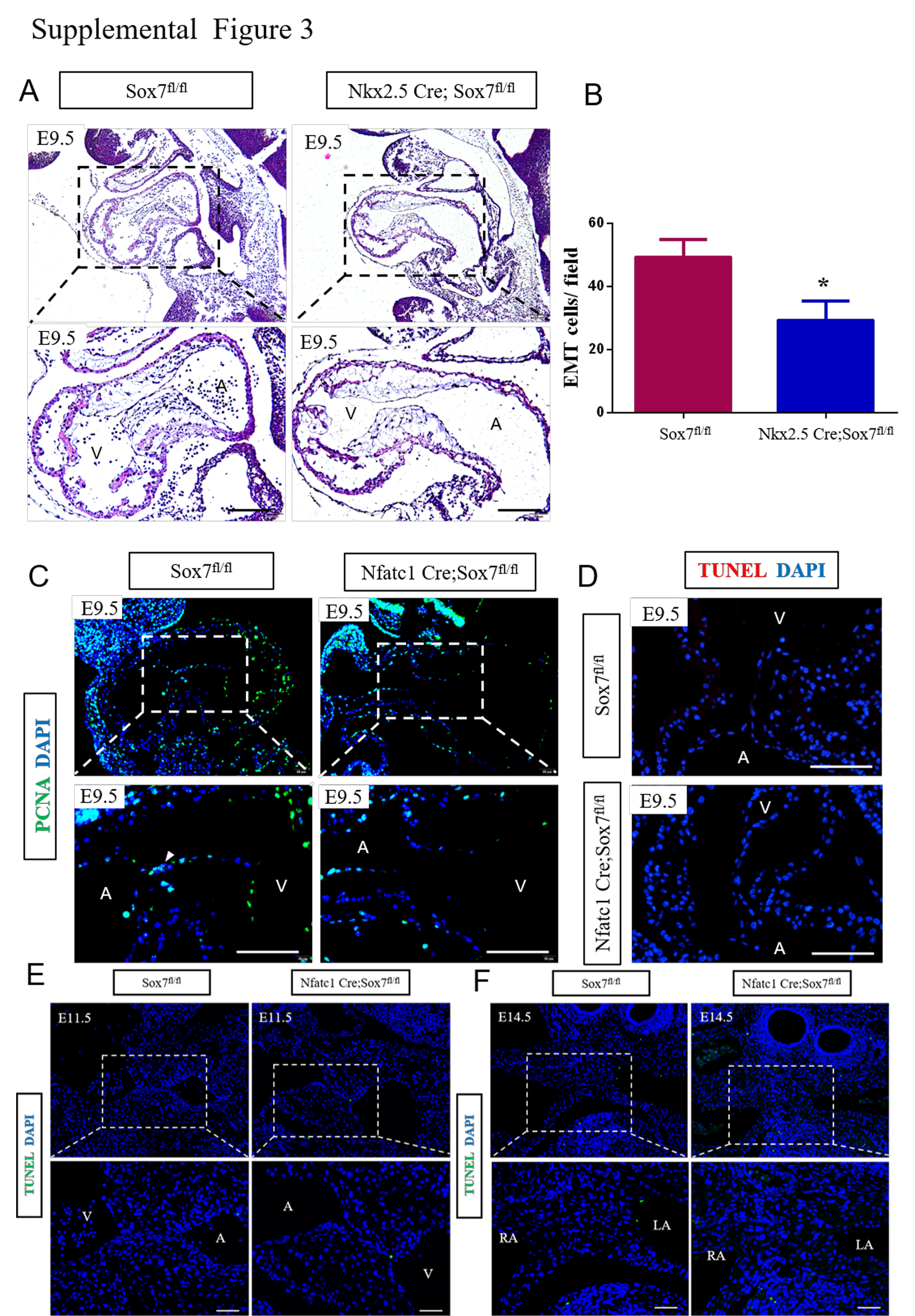

Supplement: Supplementary file 4 — Supplementary figure 3 [file 41419_2021_3658_MOESM4_ESM.tif]

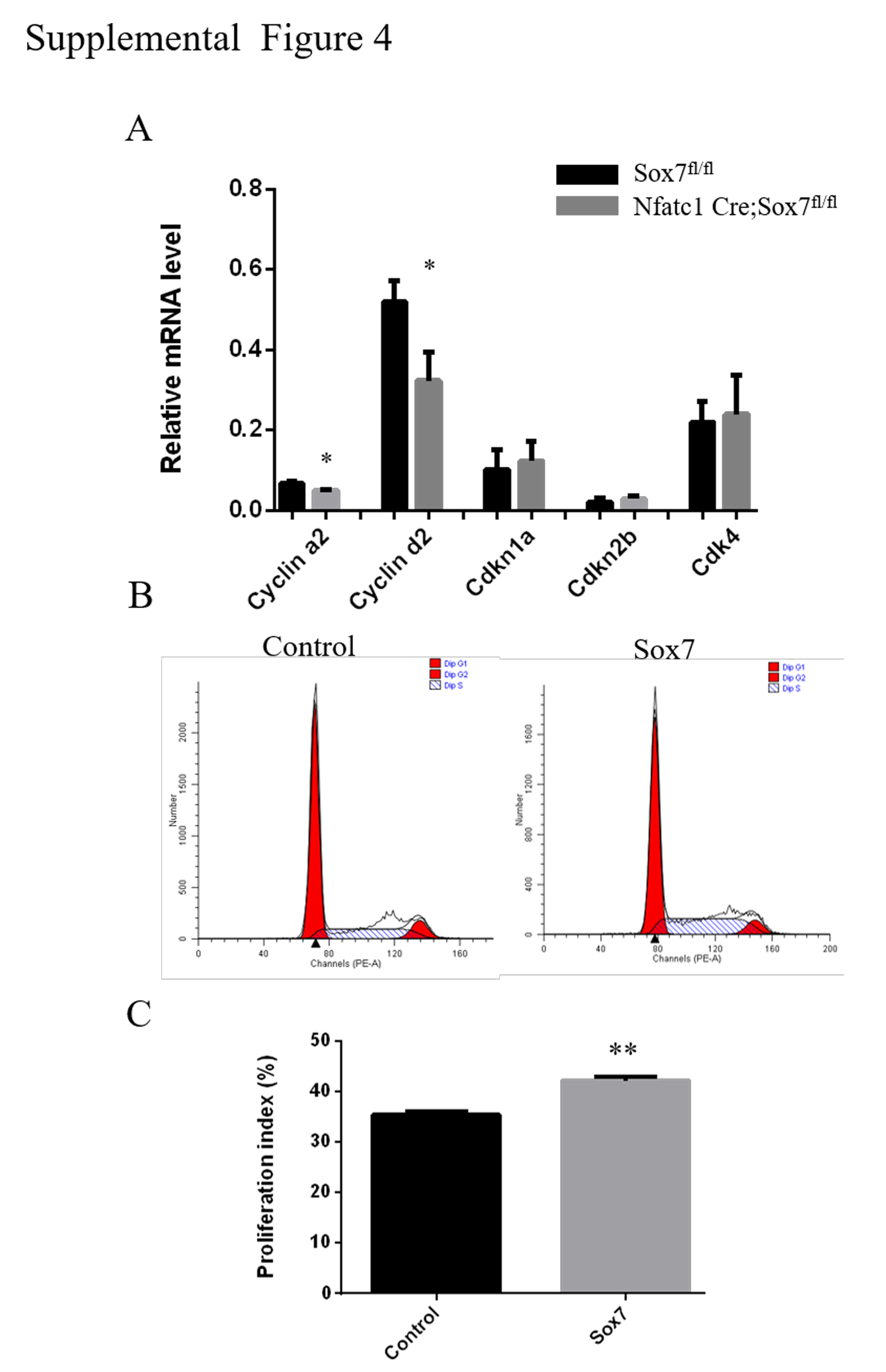

Supplement: Supplementary file 5 — Supplementary figure 4 [file 41419_2021_3658_MOESM5_ESM.tif]

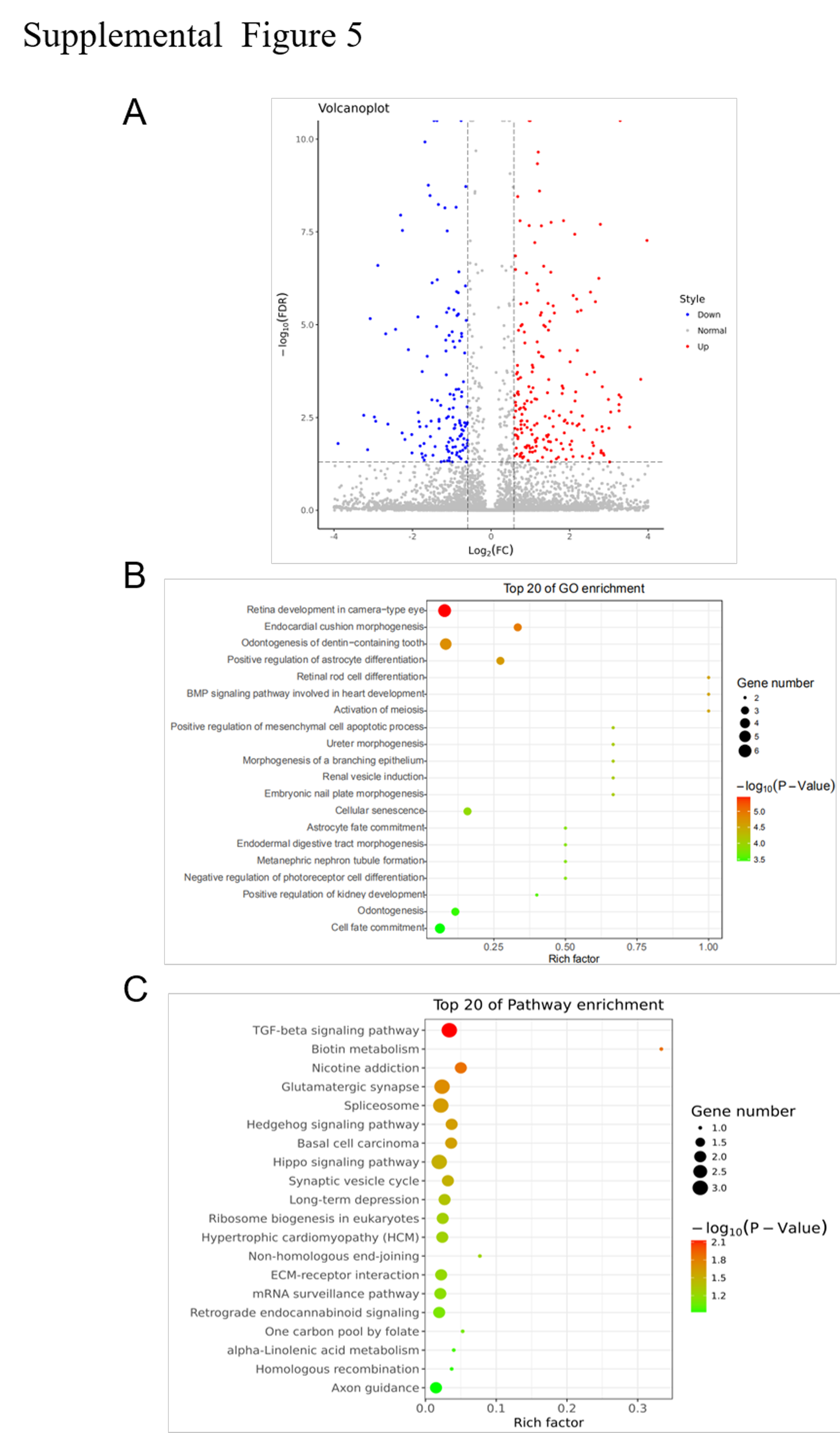

Supplement: Supplementary file 6 — Supplementary figure 5 [file 41419_2021_3658_MOESM6_ESM.tif]

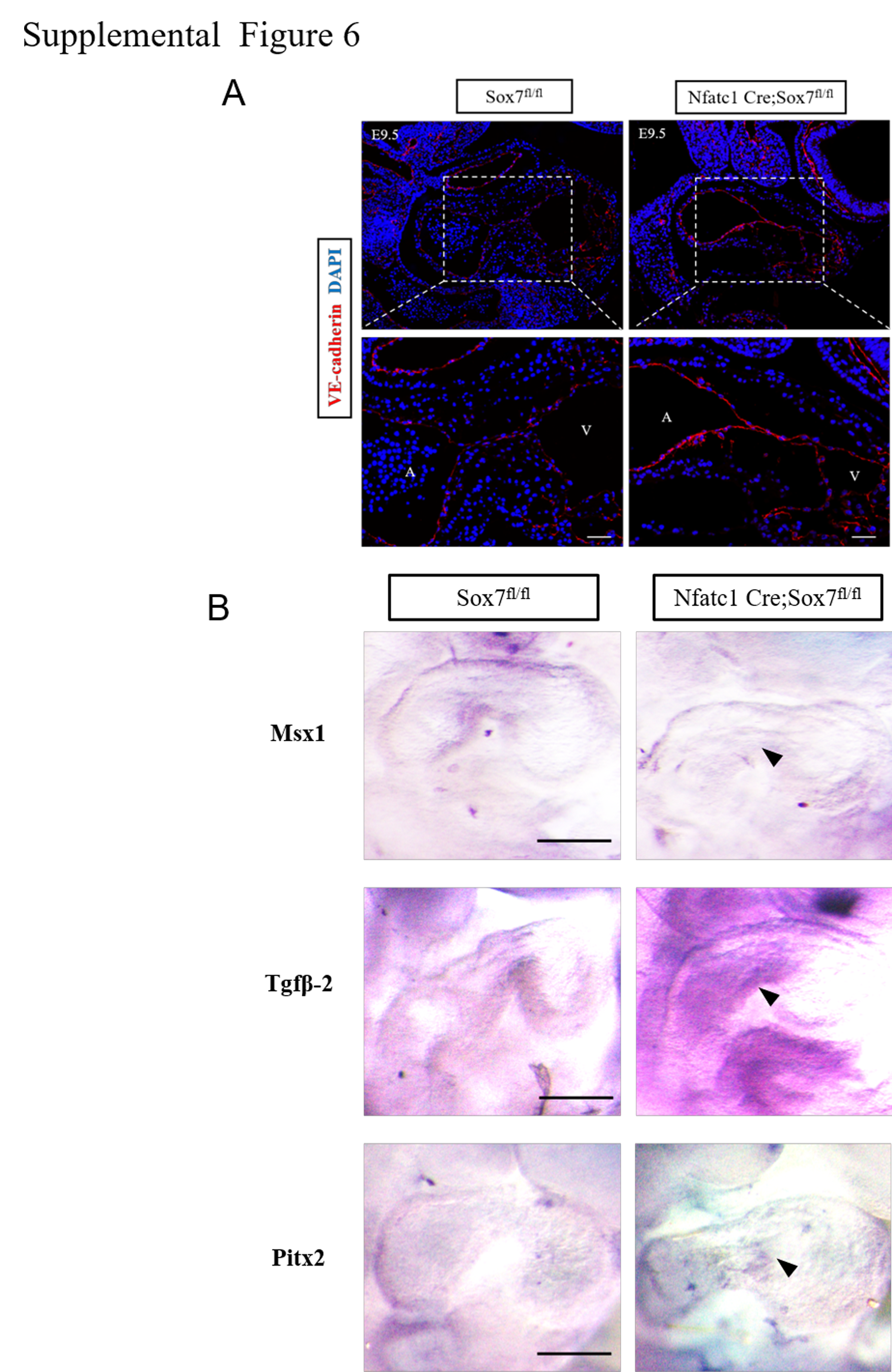

Supplement: Supplementary file 7 — Supplementary figure 6 [file 41419_2021_3658_MOESM7_ESM.tif]

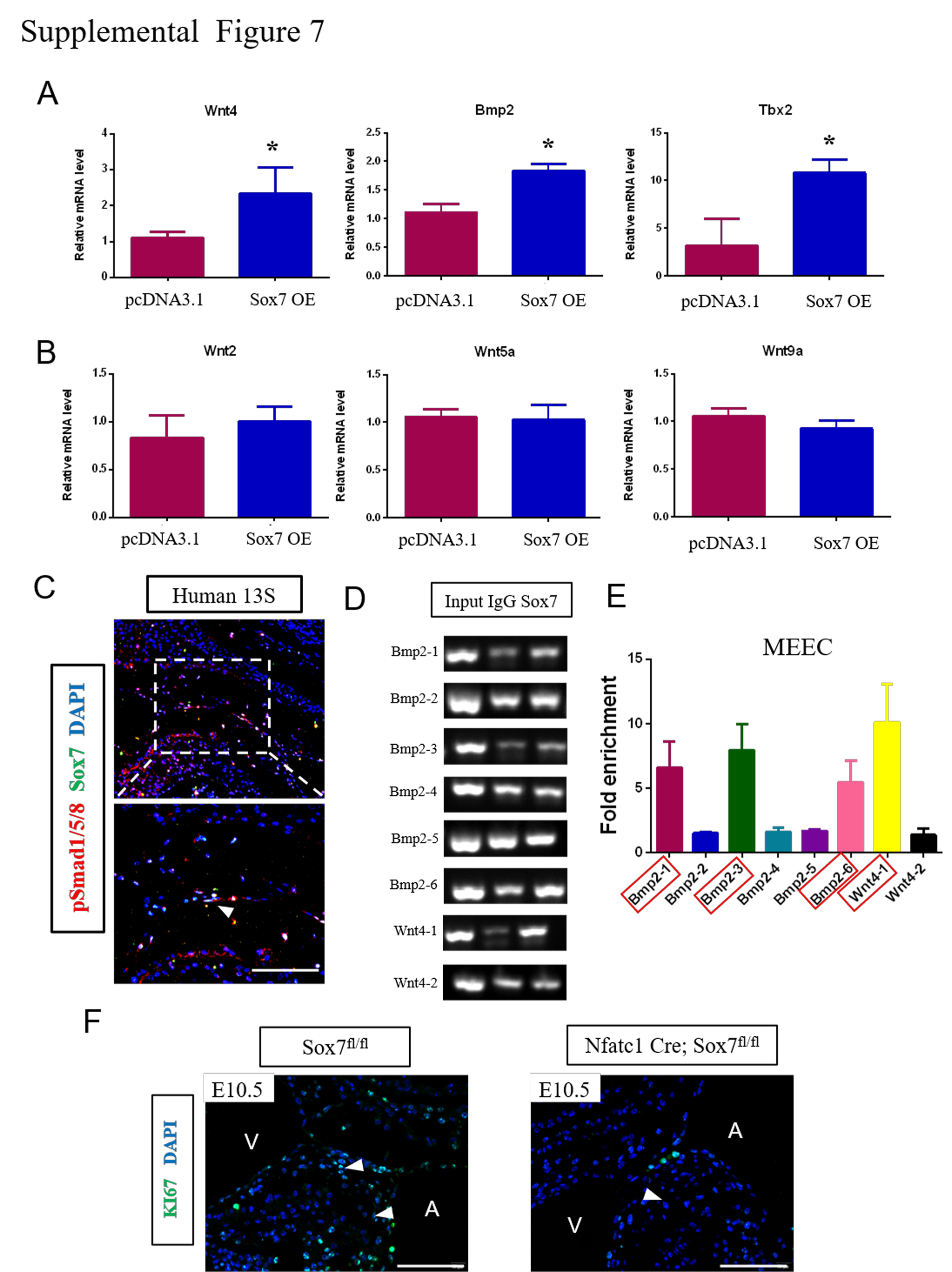

Supplement: Supplementary file 8 — Supplementary figure 7 [file 41419_2021_3658_MOESM8_ESM.tif]
