# Supplementary material for: The transcription factor Sox7 modulates endocardiac cushion formation contributed to atrioventricular septal defect through Wnt4/Bmp2 signaling
Source: Cell Death Dis. 2021 Apr 12;12(4):393. doi: 10.1038/s41419-021-03658-z (PMC8041771; doi:10.1038/s41419-021-03658-z)
Supplement: Supplementary file 9 — Supplementary Tables [file 41419_2021_3658_MOESM9_ESM.docx]

**Table S1 Ten rare copy number variants identified in patients with AVSD**

| **Patient ID** | **Chromosome** | **Genomic coordinates** | **Type** | **Size (kbp)** | **Genes altered** |
| --- | --- | --- | --- | --- | --- |
| 1 | 3q26.32-q26.33 | 178,745,197-179,458,180 | DUP | 712.983 | KCNMB3,GNB4,NDUFB5,ZNF639,  ZMAT3,USP13,MFN1,MRPL47,ACTL6A,PIK3CA |
| 2 | 8p23.1 | 8,098,153-10,588,092 | DEL | 2489.939 | PRSS55,TNKS,RP1L1,PPP1R3B,SGK223,CLDN23,MFHAS1,ERI1,C8orf74,  SOX7,MSRA,FAM86B3P |
| 3/4/5/6 | 9q13 | 66,499,410-67,293,768 | DUP | 794.358 | AQP7P1,PTGER4P2-CDK2AP2P2 |
| 7 | 13q14.11 | 42,005,985-42,780,319 | DUP | 774.334 | OR7E37P,VWA8,DGKH,RGCC |
| 8/9 | 21q11.2-q21.1 | 15,954,403-17,197,382 | DUP | 1242.979 | SAMSN1,USP25,NRIP1 |
| 10 | 21q21.1 | 22,370,838-22,910,378 | DUP | 539.540 | RNU6-67P,NCAM2 |
| 11 | 21q21.3 | 27,141,234-28,338,583 | DUP | 1197.349 | ADAMTS5,ADAMTS1,APP,CYYR1,  GABPA |
| 12 | 21q22.11 | 32,499,264-33,060,790 | DEL | 561.526 | SCAF4,TIAM1,SOD1 |
| 8/13 | 21q22.13-q22.2 | 38,559,418-40,670,684 | DUP | 2111.266 | DSCR9,DSCR8,KCNJ15,ERG,DSCR10,DSCR3,ETS2,TTC3,PSMG1,DSCR4,  DYRK1A,BRWD1,KCNJ6 |
| 8 | 21q22.3 | 45,858,958-46,917,076 | DUP | 1058.118 | SUMO3,POFUT2,TSPEAR,UBE2G2,  SSR4P1,TRPM2,PTTG1IP,ITGB2,COL18A1,KRTAP10-12,KRTAP10-11, LRRC3,KRTAP10-10,KRTAP12-1, KRTAP12-3,KRTAP12-2,KRTAP12-4, KRTAP10-9,KRTAP10-8,KRTAP10-7, LRRC3-AS1,KRTAP10-5,KRTAP10-4, KRTAP10-3,KRTAP10-2,KRTAP10-1, ADARB1,FAM207A,KRTAP10-6 |

**Table S2 Probe primers used to amplify genes within the 8p23.1 deletion region**

| Probe name | Forward primer | Reverse primer |
| --- | --- | --- |
| Prag1 | TGCCGTGAAGCCCACCATGA | TGCGAGACTTCGGCACTCAGG |
| Mfhas1 | CGCATGGCAAGCCATAACCCC | ACGGTGTAGTGCAGTCCAGGC |
| Msra | AGGTATGCGCCAGGGGAACG | TTGCTTGGCAGAGGTCGGGT |
| Sox7-1 | AGGGACTGGGGACACGGACT | TCCGGTTGTTCGGGTGTGTCC |
| Sox7-2 | GAAGAAGCAGGCCAAGCGGC | CTTCTCCGGCAGGGCGTTCT |
| Pinx1-1 | CCGGCGAGGGAGTTACGCAC | CTGGACCTGGGTGACTGCGG |
| Pinx1-2 | CAGCTCTGGAGGAGCCTCGTT | GGCCCTCAGGCTCGGAAAGC |
| Gata4-1 | TCCTGCTCTTCTGTACGCTGTCC | AGATTGGCCCGTCCCTGCTT |
| Gata4-2 | TGCCCCATCCATCCGCTTGA | GTCACTTGTATGTCACACCCCACA |

**Table S3 Primers used for In Situ Hybridization probes**

| Primer name | Forward primer | Reverse primer |
| --- | --- | --- |
| Bmp2 | GGCCAGCCAGGAGCG | GTGCTGGAGTTGAACCCATA |
| Has2 | CTTCCTCAGCAGCGTGAGAT | GGCAGATGCACAGTAAGGGA |
| Msx1 | CGCCATGGGCACAGATGA | CTGGGGACCACGGATAAA |
| Snai1 | CAAACCCACTCGGATGTGAAGAGA | GTGAAACAGGTGTCACCAGGACAA |
| Sox9 | ACGGAACAGACTCACATC | TTAACGACAGACAAAAAA |
| Tbx2 | TCCTGCACCCAGGACAATTC | TGACACTCAAGCCCAAGGC |
| Tbx20 | CGAGCAGCTCCTCAAACAGA | TGGGCTTGAGTTAGTCTTGTCA |
| Sox7 | tccgcgactgtgacagggtctt | tttgacgacaaggtctcggaa |
| Notch1 | GGGCTATGAATTTCACCGTGG | GTCTGACAGTCCTCATCAGCTTGAC |
| Pitx2 | AAATGGAGAAAGCGGGAA | GATGGGTCGTACATAGCA |
| Wnt4 | AACATCGCCTATGGCGTAGC | GGTCAATGTGGACAGCCAGAG |
| Tgfb2 | GAGCAGCGGATTGAACTGTATC | TCCCCCTGGCTTATTTGAGT |

**Table S4 Primers used for qPCR**

| Primer name | Forward primer | Reverse primer |
| --- | --- | --- |
| Sox7-mice | ATGCTGGGAAAGTCATGGAAG | CGTGTTCTGGTCACGAGAGA |
| Sox7-human | TCGACGCCCTGGATCAACT | CTGGGAGACCGGAACATGC |
| Wnt4-mice | AGACGTGCGAGAAACTCAAAG | GGAACTGGTATTGGCACTCCT |
| Wnt2-mice | CTCGGTGGAATCTGGCTCTG | CACATTGTCACACATCACCCT |
| Wnt5a-mice | CAACTGGCAGGACTTTCTCAA | CATCTCCGATGCCGGAACT |
| Wnt9a-mice | GGCCCAAGCACACTACAAG | AGAAGAGATGGCGTAGAGGAAA |
| Tbx2-mice | CCGATGACTGCCGCTATAAGT | CCATCCACTGTTCCCCTGT |
| Bmp2-mice | GGGACCCGCTGTCTTCTAGT | TCAACTCAAATTCGCTGAGGAC |
| Cyclin a2-mice | TGCTTTTGACTTGGCTGCAC | GCAGCTCCAGCAATGAGTGA |
| Cyclin d2-mice | GAGTGGGAACTGGTAGTGTTG | CGCACAGAGCGATGAAGGT |
| Cdk4-mice | AAGGTCACCCTAGTGTTTGAGC | CCGCTTAGAAACTGACGCATTAG |
| Cdkn1a-mice | CTTGTCGCTGTCTTGCACTC | CAATCTGCGCTTGGAGTGAT |
| Cdkn2b-mice | GCCACCCTTACCAGACCTGT | TTCAGCCAAGTCTACCGGC |
| Gapdh-human | GGAGCGAGATCCCTCCAAAAT | GGCTGTTGTCATACTTCTCATGG |
| Gapdh-mice | AGGTCGGTGTGAACGGATTTG | TGTAGACCATGTAGTTGAGGTCA |

**Table S5 Primers used for ChIP-qPCR**

| Primer name | Forward primer | Reverse primer |
| --- | --- | --- |
| Bmp2-1 | AGCTGCTTGCTAGATGTGAGAC | GTGTTTCAGGGGACTAGTCAGG |
| Bmp2-2 | ACTTCTTCCACAGGCTCCTAAA | GGAGCCATACACCGTAGACT |
| Bmp2-3 | AGGCCTCTTGCTTTATGGTG | ACTTTTGGAGATGTGACTGGATCA |
| Bmp2-4 | AGATTGTAACTACCCCCTTTCCC | GACCAGAAGCTACAGCCGA |
| Bmp2-5 | AGCAAATGCTACTCTCGGCT | TGTTCCTACTAGGTGGACCATT |
| Bmp2-6 | AGGTAGATGGATCATGTCACGC | GGAGGCTGCTGCTATTCTATTCT |
| Wnt4-1 | CAGCTCTGGAGGAGCCTCGTT | GGCCCTCAGGCTCGGAAAGC |
| Wnt4-2 | AAAACATAGGCAGAGACCGCA | GGACCCCAATCAGGTTTGCT |
